# Specifying pathway requirements for mobile outreach cancer testing before rollout: an implementation-aware Target Product Profile method

**DOI:** 10.1186/s43058-026-01041-7

**Published:** 2026-07-16

**Authors:** Patrick Kierkegaard, Bowen Su, Masood Moghul, Declan Cahill, Nicholas David James

**Affiliations:** 1 Cancer Research UK Convergence Science Centre, Imperial College London and the Institute of Cancer Research, London, UK; 2 Department of Surgery and Cancer, Imperial College London, London, UK; 3 Department of Urology, The Royal Marsden NHS Foundation Trust, London, UK; 4 Division of Radiotherapy and Imaging, The Institute of Cancer Research, London, UK

**Keywords:** Target product profile, Implementation-aware target product profile, Mobile outreach, Cancer testing, Pathway specification, Adaptation, Implementation planning, Qualitative methods

## Abstract

**Background:**

Mobile cancer-testing services can widen access by taking testing to community or workplace sites. But the test is only one part of the pathway: eligibility explanation, informed choice, privacy, documentation, result communication, referral, and follow-up move outside clinics. Unless specified before rollout, these tasks can make a service easier to attend but harder to govern. In diagnostic-test development, a Target Product Profile is an advance brief defining a test’s intended users, use setting, and minimum and preferred features. To create an equivalent brief for outreach services, we adapted that approach into an implementation-aware Target Product Profile (iTPP). The iTPP turns evidence on delivery and handover into a requirement register, separating what must be in place, what would improve quality, what depends on local set-up, and what needs further evidence.

**Methods:**

We conducted a qualitative method-development study using the Man Van mobile prostate-specific antigen (PSA) testing pathway as an exemplar, not an effectiveness evaluation. We interviewed attenders (*n* = 23), planning and delivery stakeholders (*n* = 13), and prospective stakeholders (*n* = 11). Using the Framework Method and abductive analysis, we charted interview evidence, converted delivery and handover issues into requirement statements, judged consequences of absence, and placed unresolved quantitative or operational questions in an Evidence and Decision Agenda.

**Results:**

The method produced a Man Van-derived register with 42 attributes across six clusters: outreach legitimacy; privacy and dignity; deliverability; test acceptability and performance uncertainty; counselling and next steps; and data integration with primary care. Handover requirements centred on result ownership, record integration, safety-netting, and loop closure: documented responsibility acceptance by a named receiving service when action was required. Diagnostic thresholds, referral ratios, staffing ratios, outreach pacing, unit costs, and equity effects were treated as questions for further evidence, not universal requirements.

**Conclusions:**

The iTPP is a pre-rollout method for specifying mobile outreach cancer-testing pathways before local adaptation, implementation, or evaluation. It identifies what should be preserved, what can vary by context, and what should remain open until stronger evidence is available. This PSA exemplar demonstrates the method; it does not establish service effectiveness, validate iTPP as an independent instrument, or recommend population screening.

**Supplementary Information:**

The online version contains supplementary material available at https://doi.org/10.1186/s43058-026-01041-7.

Contributions to the literature • Mobile cancer testing changes the pathway, not only the test location: invitation, informed choice, privacy, records, result communication, and handover become design and accountability tasks.

• The implementation-aware Target Product Profile adapts Target Product Profile logic, used in product and diagnostic-test development, to outreach service pathways.

• The method converts interview evidence into a register of minimum, desirable, and conditional pathway requirements before local adaptation.

• It keeps unresolved thresholds, referral ratios, staffing, costs, and equity effects as evidence questions rather than premature requirements.

• It can support adaptation, strategy selection, and prospective evaluation, but does not evaluate effectiveness or recommend screening.

## Background

Mobile outreach programmes can extend access to cancer testing by moving services into community and workplace settings and by lowering some geographic, temporal, and institutional barriers for groups not consistently reached through facility-based routes [1–3]. However, the test is not the only element that moves. A mobile testing encounter also relocates invitation, eligibility explanation, privacy protection, counselling, documentation, result communication, referral, and follow-up into a temporary, visible, and cross-organisational setting. Evidence from mobile cancer testing and ambulatory result-management literature shows recurrent vulnerabilities at the interface with usual care, including delayed follow-up after abnormal findings and failures to close the communication-action loop for test results [4–6]. Mobile outreach therefore creates a pathway-specification problem as well as an access opportunity: reach may expand while continuity, accountability, and safety remain under-specified.

The specification problem is concrete. Before rollout, teams must decide who should be invited, what makes an invitation legitimate, what information is needed for informed participation, how privacy is protected in visible venues, how counselling and documentation are delivered within mobile capacity, who owns results, where records go, and when responsibility transfers to another service. If these conditions remain tacit, local adaptation and implementation strategy selection begin from an underspecified pathway. Teams may then preserve, vary, or omit safeguards without an explicit basis for deciding which differences matter.

In implementation terms, the implementation object is the service, practice, or pathway whose adoption, delivery, adaptation, or sustainment is being planned. Here, that object is not the diagnostic assay alone, but the configured outreach pathway: invitation, eligibility explanation, booking or walk-up access, privacy controls, on-site testing, counselling, documentation, result communication, referral, record integration, and handover [7]. Implementation strategies can then support adoption, delivery, adaptation, or sustainment of that specified pathway [8]. This distinction matters because the pathway can fail at the interfaces between invitation, attendance, counselling, result communication, record integration, referral, and responsibility transfer even when the assay is available and the outreach offer is acceptable.

Target Product Profiles (TPPs) come from product and diagnostic-test development. Their practical role is to define the development target in advance: who the product or test is for, how and where it will be used, which features are minimum requirements, which are preferred, and what evidence remains missing [9–11]. Service-oriented TPP adaptations extend this discipline toward workflow and delivery characteristics [12]. The purpose is not to treat outreach services as products, but to make pathway requirements explicit before local adaptations begin.

For mobile outreach, the object is the pathway rather than the test alone. The parallel question is not only “What should this test be able to do?” but “What must be in place for testing outside usual clinical sites to be acceptable, feasible, safe, and governable?” We use implementation-aware Target Product Profile, or iTPP, for this pathway-level adaptation. The iTPP produced in this study comprises the requirement register, the evidence trail behind each requirement, and decision categories that separate minimum safeguards, desirable requirements, conditional requirements, and questions needing further evidence. Minimum requirements are conditions whose absence would plausibly create harm, predictable delivery failure, inequitable exclusion, or unsafe handover. Desirable requirements strengthen quality, acceptability, or efficiency. Conditional requirements are triggered by configurations such as walk-in access, high-footfall venues, digital booking, or constrained privacy space. Thresholds, staffing ratios, costs, referral ratios, and equity effects are placed in an Evidence and Decision Agenda rather than written into the register as universal requirements.

Existing implementation, design, safety, and adaptation approaches inform this problem but answer different lifecycle questions. Determinant frameworks and implementation outcome taxonomies classify barriers, facilitators, mechanisms, and success criteria [13–16]. TIDieR, StaRI, and implementation strategy specification guidance improve reporting once an intervention, study, or strategy package has been defined [8, 17, 18]. Implementation Mapping and tailored blueprints support strategy development for a defined intervention or pathway [19, 20]. Human-centred design, human factors, journey mapping, service blueprinting, and safety-case approaches can reveal needs, workflow constraints, touchpoints, interface frictions, and assurance questions [21–25]. Adaptation frameworks and core-function/core-form approaches help document modifications and distinguish what should be preserved from local forms [26–30]. What remains less specified is the earlier decision point addressed here: how to identify candidate minimum pathway requirements, conditional requirements, and evidence-needed parameters before local adaptation or strategy selection. iTPP does not treat interview data as identifying definitive universal core functions; criticality requires local stress-testing and recalibration.

We therefore developed and demonstrated iTPP as a prospective pathway-specification method for mobile outreach cancer testing. Its intended users are implementation researchers, clinical pathway leads, service designers, commissioners, cancer alliances, and evaluation teams defining an outreach pathway before local adaptation, strategy selection, or prospective evaluation. We use a mobile prostate-specific antigen (PSA) testing pathway linked to National Health Service (NHS) diagnostic services as the empirical exemplar. The aim is not to evaluate PSA testing, make a screening recommendation, or validate iTPP as an independent instrument. The transferable contribution is the translation procedure, requirement structure, and decision logic, not the substantive content of any single PSA pathway register.

## Methods

### Study design and analytic aim

We conducted a qualitative method-development study using the Man Van mobile PSA pathway as an empirical exemplar, not as an effectiveness evaluation. The analytic task was to specify the end-to-end outreach pathway as the implementation object: invitation, attendance, on-site testing, counselling, documentation, result communication, referral, and handover [7]. We analysed two evidence streams. The Pilot stream captured realised delivery and mobile-to-usual-care transition constraints in Man Van. The Prospective stream elicited anticipatory requirements for future pathway variants and alternative non-invasive modalities and was not used to infer observed Man Van performance.

Interview data were analysed through a staged translation procedure that converted material about delivery, counselling, documentation, result communication, referral, and handover into pathway requirements. Participants were not asked to define an iTPP; the author team derived the iTPP analytically from charted evidence. The procedure produced three outputs: the requirement register, the Evidence and Decision Agenda, and a bounded transition-focused determinant crosswalk. The register was the main pathway-specification output. The Evidence and Decision Agenda captured consequential parameters that could not be fixed from interview data without false precision, and the crosswalk connected transition-related risks with candidate monitoring signals.

### Empirical exemplar

The empirical exemplar was Man Van, a community-based mobile unit delivering PSA testing linked to NHS diagnostic pathways across six London boroughs between 2022 and 2024, with on-unit counselling, PSA testing, results communication, and referral where indicated [31].

### Participants, sampling, and recruitment

We analysed two evidence streams. The Pilot stream comprised Man Van attenders (*n* = 23) and planning or delivery stakeholders directly involved in the pathway (*n* = 13). The Prospective stream comprised general practitioners (GPs; *n* = 4), patient participants (*n* = 5), and patient representatives (*n* = 2). The total sample was 47. Sampling was purposive and sought variation across pathway roles, interfaces, deployment site, ethnicity, and area-level deprivation where feasible. Participant characteristics are summarised in Table 1.

Eligibility criteria were defined a priori. Attenders had used Man Van, completed the post-service evaluation questionnaire, and consented to future contact. Planning or delivery stakeholders were involved in Man Van planning, mobilisation, delivery, pathway integration, or governance. Prospective participants had relevant expertise or experience in mobile outreach design, follow-up management, or patient-facing acceptability issues relating to mobile delivery and non-invasive modalities.

Sampling continued until specification sufficiency within each stream, defined as the point at which additional interviews no longer introduced new requirement-level concepts or materially changed statement-level criticality beyond editorial refinement. Pilot-stream attenders were recruited from consenting post-service questionnaire respondents; planning and delivery stakeholders through professional networks; and Prospective participants through the South London Clinical Research Network and cancer-charity advertisements. The Prospective stream was analysed separately because it informed future-facing requirements rather than observed Man Van performance.

**Table 1:** Participant characteristics by evidence stream (*N* = 47)

Characteristic	Total (*N* = 47)	Man Van attenders, Pilot stream (*n* = 23)	Planning/delivery stakeholders, Pilot stream (*n* = 13)	Prospective stakeholders, prospective stream (*n* = 11)
**Age**,** median (range)**,** years**	61 (32–88)	64 (52–74)	48 (37–65)	60 (32–88)
**Age categories**,** No. (%)**				
< 40 years	7 (14.9%)	0 (0%)	4 (31%)	3 (27%)
40–49 years	5 (10.6%)	0 (0%)	4 (31%)	1 (9%)
50–59 years	8 (17.0%)	6 (26%)	1 (8%)	1 (9%)
60–69 years	18 (38.3%)	14 (61%)	0 (0%)	4 (36%)
70 + years	4 (8.5%)	3 (13%)	0 (0%)	1 (9%)
Not reported	5 (10.6%)	0 (0%)	4 (31%)	1 (9%)
**Gender**,** No. (%)**				
Male	33 (70%)	23 (100%)	6 (46%)	4 (36%)
Female	14 (30%)	0 (0%)	7 (54%)	7 (64%)
**Ethnicity**,** No. (%)**				
White (British/Irish/Euro)	27 (57.4%)	18 (78%)	2 (15%)	7 (64%)
Black (African/Caribbean)	1 (2.1%)	1 (4%)	0 (0%)	0 (0%)
Asian / Asian British	4 (8.5%)	1 (4%)	2 (15%)	1 (9%)
Mixed / Multiple Heritage	3 (6.4%)	2 (9%)	0 (0%)	1 (9%)
Other	1 (2.1%)	1 (4%)	0 (0%)	0 (0%)
Not reported / declined	11 (23.4%)	0 (0%)	9 (69%)	2 (18%)
**Occupation (proxy for socioeconomic position)**,** No. (%)**				
Professional / Managerial (e.g. Director, Head Teacher, Solicitor, GP, Lawyer)	16 (34%)	14 (61%)	2 (15%)	NA
Skilled Non-Manual / Technical (e.g. Buyer, Photographer, H&S Consultant)	10 (21%)	6 (26%)	4 (31%)	NA
Routine / Manual / Service (No manual workers identified in attender sample)	1 (2%)	0 (0%)	1 (8%)	NA
Not reported / not collected	20 (43%)	3 (13%)	6 (46%)	11 (100%)
**Man Van record-linked characteristics (attenders only)**				
Man Van site of attendance, No. (%)	NA	Ealing Townhall 10 (43%); Balham GP 4 (17%); Ikea 3 (13%); Chelsea FC 3 (13%); Bridge Park 3 (13%)	NA	NA
**Index of Multiple Deprivation (IMD) quintile of residence (Q1 = most deprived)**,** No. (%)**	NA	Q1 3 (13%); Q2 1 (4%); Q3 6 (26%); Q4 9 (39%); Q5 3 (13%); missing 1 (4%)	NA	NA

Note: Data are n (%) unless otherwise indicated; percentages are column percentages and may not sum to 100 because of rounding. Occupation categories were derived from self-described job titles and are used as a pragmatic proxy for socioeconomic position. For attenders, site of attendance and IMD quintile were extracted from programme records to describe recruitment context. NA indicates not applicable or not collected

### Data collection

Semi-structured topic guides were co-designed with the Man Van clinical team and refined iteratively. Guides covered experiences of attending or delivering the service, legitimacy and trust, privacy and acceptability, delivery constraints, results communication, follow-up, and requirements for wider implementation.

Interviews were conducted between April 2024 and April 2025 by MM (male; consultant urologist; MD/PhD), BS (female; public health researcher; MD/PhD), and PK (male; qualitative health services researcher; PhD-MPH). Interviews lasted about 60 min and were conducted mainly online, with one telephone and one face-to-face attender interview. Interviewers had no prior relationship with attenders. Some planning and delivery stakeholders were known professionally to MM; when MM participated, BS or PK led the interview. Participants were reminded that critical views were welcome and that participation would not affect care or services.

Interviews were audio-recorded with consent, and participants consented to the use of de-identified quotations. Artificial intelligence (AI)-assisted transcription software (Buzz) was used only to generate draft transcripts. Transcripts were manually checked against recordings to correct errors and remove identifiers [32], and de-identified transcripts were stored on encrypted institutional servers.

### Analytic procedure for translating evidence into pathway requirements

Interview data were analysed through a staged translation procedure that produced three outputs: an iTPP register, an Evidence and Decision Agenda, and a bounded transition-focused determinant crosswalk. The register was the main pathway-specification output. The Evidence and Decision Agenda captured consequential parameters that could not be fixed from interview data without false precision. The crosswalk connected transition-related risks with candidate monitoring signals.

Analysis followed the Framework Method stages of familiarisation, coding, framework development, indexing, charting, and interpretation in NVivo v15 [33]. BS and PK led coding and charting, with MM contributing to interpretive adjudication. The abductive framework was seeded from medical-test Target Product Profile domains and broader Target Product Profile guidance, then expanded inductively to capture delivery frictions, service-envelope constraints, and mobile-to-usual-care transition risks [9–11, 34]. BS and PK double-coded a purposive 20% subset spanning participant groups and both evidence streams; differences were resolved before full indexing and charting.

The analytic hierarchy was excerpt to coded segment to charted issue to attribute to requirement statement to requirement cluster. Attributes were assigned unique identifiers; modality was assigned to the statement nested within an attribute rather than to the attribute label itself; and each statement was linked to supporting excerpts and evidence provenance: Pilot only, Prospective only, or Both streams. Box 1 summarises the translation rules.

**Box 1:** How stakeholder evidence was translated into pathway requirements

1. **Define the pathway boundary**: Specify the test or service, intended users, delivery setting, pathway start and end points, excluded claims, and receiving services. 2. **Chart stakeholder evidence**: Chart excerpts against pathway actions, actors, settings, handover points, failure risks, and affected groups. 3. **Translate charted issues into requirement statements**: Write requirements as delivery or handover conditions, not as themes. Assign each statement to a cluster and unique attribute ID. 4. **Assign consequence-based criticality**: Use MUST when absence would plausibly create harm or predictable delivery failure under realistic operating conditions; SHOULD when desirable but not necessary to prevent plausible harm or failure; and IF-WHEN when the requirement is triggered by a specific configuration or context. MUST denotes consequence-based criticality within this study, not a legal duty or universal standard. 5. **Separate evidence-needed parameters**: Do not embed numeric thresholds, staffing ratios, costs, or equity effects as universal requirements unless they can be specified defensibly from the evidence. Record unresolved parameters in the Evidence and Decision Agenda. 6. **Record rationale and provenance**: Link each statement to evidence provenance, record the criticality rationale, and document where analytic judgement rather than direct quotation shaped the final requirement statement.	

A worked trace illustrates the logic. Participants described walk-in access as desirable but constrained by pre-registration, staffing, space, and privacy. This was translated into linked requirements specifying whether the service is appointment-based, walk-in, or mixed, and aligning queue management, eligibility checks, staffing, and privacy controls accordingly. Because walk-in access became necessary only under some operating configurations, the related statement was assigned to the IF-WHEN layer rather than treated as a universal minimum. Additional worked traces are provided in Additional file 2.

PK initially assigned statement-level modalities, and BS independently reviewed assignments blind to PK’s grading. Disagreements were resolved through discussion and recorded in the modality decision log. MUST, SHOULD, and IF-WHEN designations denote consequence-based criticality rather than legal obligation and are intended to be stress-tested and recalibrated locally.

To avoid false precision, numeric thresholds, staffing ratios, unit costs, referral ratios, and equity effects were not embedded as universal requirements unless defensible from the interview corpus. Consequential but non-specifiable parameters were recorded in the Evidence and Decision Agenda for later modelling, evaluation, or local configuration [35, 36]. After the register had been constructed, transition-relevant segments were labelled descriptively with Consolidated Framework for Implementation Research (CFIR) 2.0 constructs and mapped to corresponding iTPP requirement anchors and candidate monitoring signals [15, 16]. This supplementary crosswalk was limited to the mobile-to-usual-care transition and did not determine register structure, requirement wording, or modality assignment.

Additional file 2 contains the register, Evidence and Decision Agenda, determinant crosswalk, data dictionary, worked exemplars, and Worksheets B1–B6. The worksheets support pathway-boundary definition, evidence charting, requirement translation and criticality assignment, evidence-needed parameter separation, local adaptation recording, and prospective recalibration. They are guidance materials for replication and testing, not a validated instrument.

**Fig. 1:**
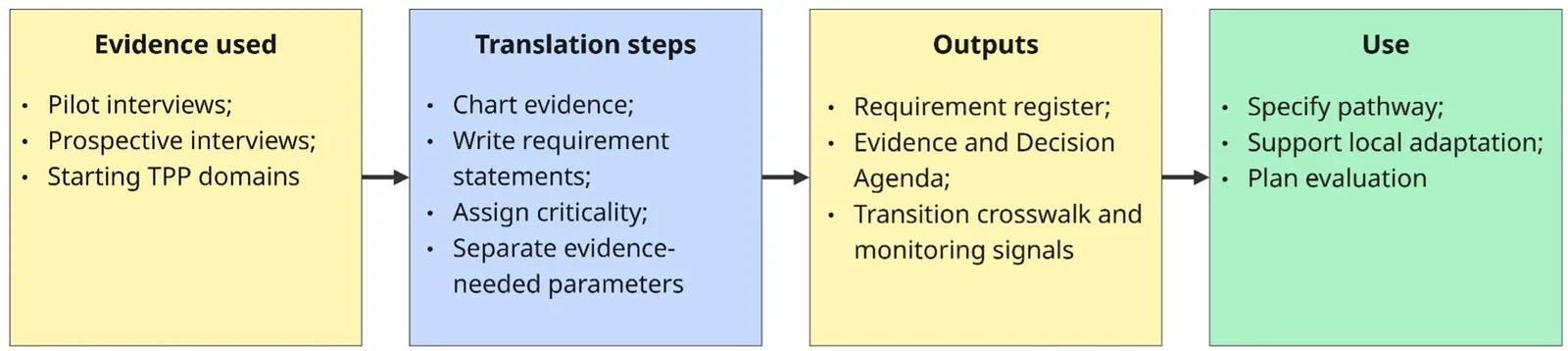
Evidence-to-requirement translation: inputs, steps, outputs, and intended uses. Note The mobile PSA pathway was the empirical exemplar. The transferable contribution is the translation procedure, requirement structure, and decision logic, not the substantive content of any single register. The figure does not imply an effectiveness evaluation, external validation of the method, or a recommendation for population screening

Figure 1 summarises the evidence inputs, translation steps, outputs, and intended uses of the method.

### Reflexivity and rigour

A multidisciplinary team supported interpretive challenge throughout analysis. Clinical expertise informed assessment of pathway safety, escalation, and follow-up risks; public health expertise informed equity-sensitive interpretation of access and delivery; and implementation science expertise informed specification logic, pathway design, and deliverability. Rigour was strengthened through comparison across participant roles and evidence streams, double-coding of a purposive transcript subset, consensus resolution, and an audit trail spanning framework iterations, translation decisions, requirement statement versions, and modality logs.

### Ethics and reporting

The Man Van project received ethical approval from Wales Research Ethics Committee 6 (REC reference 22/WA/0113; protocol CCR5573; IRAS project ID 304320). All participants provided informed consent before interview. The use of automated draft transcription was described in the approved protocol. Audio files were handled in accordance with the approved protocol, and transcript retention followed institutional data-retention policies. A steering group of patient representatives from The Royal Marsden NHS Foundation Trust reviewed the study protocol, interview guides, and participant-facing materials. Reporting follows the Consolidated Criteria for Reporting Qualitative Research [37]; the completed checklist is provided as Additional file 1.

## Results

### Pathway-specification outputs

The analysis produced a Man Van-specific pathway specification with three linked outputs: the iTPP register, the Evidence and Decision Agenda, and a bounded transition-focused crosswalk linking interface risks, iTPP attributes, and candidate monitoring signals.

The register contained 42 attributes across six clusters. Attributes were expressed as one or more auditable requirement statements at Minimum (MUST), Optimal (SHOULD), and/or Conditional (IF-WHEN) levels. MUST denotes consequence-based criticality, not a statutory requirement or universal standard. Evidence provenance was recorded as Pilot only (*n* = 6), Prospective only (*n* = 11), or Both streams (*n* = 25). Prospective-only attributes represented anticipatory requirements for future pathway variants rather than observed Man Van performance.

The clusters spanned the on-unit clinical-service envelope (RC1-RC5) and the mobile-to-usual-care transition (RC6). Box 2 summarises attribute distribution by cluster and provenance; Table 2 shows selected Minimum statements; and the full register and Evidence and Decision Agenda are provided in Supplementary Tables S1 and S2.

**Table 2:** Example Minimum (MUST) requirements from the pathway requirement register

Cluster	iTPP attribute	Minimum (MUST) requirement statement	Evidence provenance
RC1	Rationale and eligibility clarity	Provide a clear, plain-language rationale for why the test/service is being offered and who it is intended for.	Prospective stream
	Legitimacy and trust cues	Provide visible legitimacy and trust signals (NHS/clinical sponsorship, transparent governance, privacy assurances, clear branding) so the service is perceived as safe and credible.	Both
	Policy- and governance-compatible invitation channel	Provide at least one trusted invitation route compatible with applicable policy and local governance that supports informed choice and does not require a GP appointment, for example an opt-in, governance-approved GP-endorsed message where permitted, or an equivalent trusted channel, with a clear call-to-action.	Both
	Multi-channel outreach (avoid GP-text only)	Avoid single-channel outreach; plan for at least one non-GP route to reach people who do not respond to GP text invitations.	Pilot stream
RC2	Waiting, privacy and visibility	Protect privacy for conversations and sample collection, and manage waiting so people are not visibly exposed or embarrassed.	Both
	Access model (appointment vs. walk-in)	Specify whether the service is appointment-based, walk-in, or mixed, and provide basic queue/wait management to protect privacy and throughput.	Both
	Booking workflow (QR/online self-booking)	Provide a clear booking route that does not depend on digital access (for example phone or assisted booking) and that supports basic eligibility checks.	Both
	On-site experience (comfort and perceived quality)	Ensure the on-site environment is professional, clean and respectful, so the mobile offer is perceived as legitimate healthcare rather than a temporary or informal offer.	Both
RC3	Operational intensity and staffing burden	Design the delivery workflow to be feasible with realistic staffing; avoid labour-intensive steps as the default operating mode.	Both
	Partnership and coordination overhead	Establish a partnership model (integrated care board, public health, clinical provider, and community host) with clear roles, responsibilities and escalation routes.	Both
	Funding model and sustainability	Specify how the service could be sustained beyond a one-off pilot, including funding and governance arrangements.	Both
	Downstream workload management	Define who owns results and who is responsible for follow-up; do not default to unplanned GP workload without resourcing and explicit agreements.	Prospective stream
RC4	Procedure clarity (avoid feared procedures)	Provide a clear explanation of the on-site process (what happens in the van) to address anticipatory anxiety and procedural misconceptions.	Pilot stream
	Accuracy, false positives and acceptability	Be explicit about test limitations and threshold choices (false positives/negatives); avoid implicit performance claims in outreach and consent materials.	Both
	Performance thresholds (evidence-needed field)	Define intended use, state the provisional performance hypothesis, and mark numeric thresholds as evidence-needed until supported by diagnostic accuracy evidence, modelling, or consensus review.	Prospective stream
	Acceptability: non-invasive testing and invasive follow-up	Treat acceptability as dependent on downstream consequences; specify referral criteria and follow-up pathways to minimise unnecessary invasive procedures and avoid avoidable harm.	Both
RC5	Counselling and interpretation capability	Ensure access to counselling/interpretation for results with significant implications (on-site or tightly linked), so uncertainty and next steps can be managed safely.	Both
	Results communication (timing and method)	Define how results will be communicated (method, timeframe) and ensure confidentiality and accessibility.	Both
	Next steps and timelines	Provide a clear description of what happens after testing (results pathway, referral options, expected timelines, and contact routes).	Prospective stream
	Safety-netting and escalation	Provide safety-netting instructions and escalation routes for concerning symptoms or delays, independent of the mobile visit.	Both
RC6	Result ownership and GP workload	Specify ownership and responsibility for acting on results within the mobile‑testing pathway until loop closure is confirmed, where loop closure is defined as positive confirmation of transfer of clinical responsibility to a named receiving provider/service/team, evidenced in an auditable way (not merely the transmission of information). Do not transfer workload to GPs without explicit agreement and resourcing.	Both
	Data and record integration	Ensure encounter data and results are captured and accessible for ongoing care (for example can be communicated to the GP record) with appropriate data governance, including documentation of result ownership and (where onward action is required) whether loop closure has occurred.	Both
	Primary care access friction	Design follow-up assumptions acknowledging that access to GP appointments may be constrained; do not assume rapid GP follow-up as the default route.	Both
	Alignment with screening/referral pathways	Map how mobile testing outcomes connect to existing screening/referral guidance and local services to prevent fragmentation.	Prospective stream

Note: MUST denotes consequence-based criticality in this study, not a legal duty or universal standard. Modality is assigned to requirement statements rather than to the attribute label alone. The table shows selected Minimum statements; the full register, including Optimal and Conditional statements, is provided in Supplementary Table S1

**Box 2:** Requirement attributes by cluster and evidence provenance

Requirement cluster	*n* (Pilot / Both / Prospective)
RC1: Outreach, invitation and legitimacy	10 (3/6/1)
RC2: Minimising friction while protecting privacy and dignity	6 (0/6/0)
RC3: Deliverability, staffing, and sustainability	6 (0/4/2)
RC4: Test modality acceptability and performance thresholds	8 (2/3/3)
RC5: Information, counselling and next-steps pathways	7 (1/3/3)
RC6: Data integration and primary care alignment	5 (0/3/2)
Total	42 (6/25/11)

### Cross-cluster tensions shaping minimum safeguards

Three recurrent tensions shaped the MUST layer: trust and legitimacy outside routine clinical settings, privacy-visibility trade-offs in high-footfall environments, and coupling of access models to counselling, documentation, escalation, and follow-up capacity. Table 2 summarises the associated integrity risks and minimum safeguards.

**Table 3:** Structural tensions in mobile outreach testing and the MUST-level safeguards specified in the iTPP

Structural tension (what makes mobile delivery fragile)	Integrity risk (what can fail)	MUST‑level safeguard specified in the iTPP (cluster anchors)	Audit or evaluation signal (what shows the safeguard is operating)	Candidate implementation outcome or pathway consequence
Trust outside clinical buildings	Reach failure and skewed participation when invitations are screened as scams or the offer is perceived as illegitimate.	Verifiable clinical sponsorship and identifiable governance sponsor; authenticated invitation route plus non‑digital access route; clear rationale and eligibility messaging (RC1). Linked iTPP attributes: Legitimacy and trust cues; Policy- and governance-compatible invitation channel; Multi-channel outreach (avoid GP-text only); Rationale and eligibility clarity	Invitation provenance is verifiable; uptake and attendee profile are monitored against stated targeting intent; non‑digital route documented and used.	Reach / penetration; acceptability
Visibility versus privacy in community venues	Privacy breaches, reduced disclosure, and reduced acceptability when entry, waiting, or conversations are exposed in high‑footfall settings	Privacy‑by‑design layout and workflow controls; managed waiting and discrete flow; explicit access model that avoids unmanaged queues (RC2, linked to RC1 discovery cues). Linked iTPP attributes: Waiting, privacy and visibility; Access model (appointment vs. walk-in); On-site experience (comfort and perceived quality); On-site discovery cues (signage/wayfinding).	Pre‑session privacy readiness check completed; recorded mitigations for layout and acoustics; incidents or near‑misses logged; patient‑reported privacy acceptability captured.	Acceptability; appropriateness; feasibility
Demand versus on-unit capacity	Compressed counselling and documentation, visible queues, and error‑prone workflow when outreach and booking outpace staffing and space	Capacity‑coupled outreach and booking; workflow deliverability specified against realistic staffing; explicit walk‑in policy aligned to prerequisites (RC3, with RC5 counselling implications). Linked iTPP attributes: Operational intensity and staffing burden; Booking workflow (QR/online self-booking); Access model (appointment vs. walk-in); Opportunistic walk-up engagement.	Session‑level capacity plan documented; demand surges trigger throttling rules; queue visibility incidents tracked; counselling and documentation completeness audited.	Feasibility; fidelity; unintended workload transfer

Notes: MUST denotes consequence-based minimum criticality, not a statutory requirement. Integrity refers to reliable completion of intended on-unit and onward pathway steps, including follow-up for abnormal or uncertain results. Supplementary Table S3 crosswalks Table 2 safeguards to S1 attribute IDs

### Cluster-level requirement content

The six clusters specified where mobile outreach required explicit delivery or handover conditions rather than themes alone.

#### RC1: Outreach, invitation, and legitimacy

RC1 specified legitimacy cues, trusted invitation routes, and clear rationale and eligibility messaging. Attenders described familiar NHS or GP-linked channels as reducing suspicion, while stakeholders described generic outreach as liable to miss intended groups unless engagement was tailored locally. One attender linked confidence in the offer to recognisable sponsorship:

It’s useful to know it’s coming from the same source and the source I recognise… The branding of the van, I think, helped. (MV_P10; Attender; Pilot stream)

A delivery stakeholder described the need for community-specific outreach:

It is [about] knowing what works well for that community and even within the black communities themselves.” (MV_S07; Delivery stakeholder; Pilot stream)

#### RC2: Minimising friction while protecting privacy and dignity

RC2 specified privacy-by-design layout, managed waiting, and access models that protected dignity in visible community venues. Attenders and delivery stakeholders described privacy as vulnerable when acoustics, entry flow, and waiting arrangements were improvised or exposed. One delivery stakeholder described poor soundproofing:

We had a tiny little waiting room with a folding door but unfortunately the soundproofing wasn’t very good. If there was a second patient who came, he would have to wait in the waiting area for patients’ confidentiality. (MV_S03; Delivery stakeholder; Pilot stream)

An attender described the exposure associated with moving from a private consultation back into a public environment:

I didn’t realise that when I came out, it was like, ‘Oh, right’. That’s a slight lack of privacy. From that point of view. (MV_P04; Attender; Pilot stream)

#### RC3: Deliverability, staffing, and sustainability

RC3 specified capacity-coupled outreach and booking, workable staffing, partnership roles, sustainability, and downstream workload management. Delivery stakeholders described counselling, sampling, documentation, and coordination as constraining throughput when invitation volume exceeded staff and space. One stakeholder described matching outreach intensity to operational capacity:

Once we started sending texts to the GP surgery, you got a pretty reliable take up rate per output of text. That allowed us to really quite precisely tailor the workflow coming through what somebody’s got to face to the available manpower, staffing, whatever in the van. (MV_S04; Delivery stakeholder; Pilot stream)

Stakeholders also described short-term funding as a source of discontinuity:

The problem with the NHS is that we it’s all short term. Contracts and short-term interventions get lots of interest and then you just have to stop it. (MV_S05; Delivery stakeholder; Pilot stream)

#### RC4: Test modality acceptability and performance thresholds

RC4 specified procedure explanation, explicit handling of performance uncertainty, and escalation criteria aligned to downstream consequences. Participants described modality acceptability as inseparable from what a result could trigger, rather than as convenience alone. One stakeholder described the appeal of lower-burden modalities for men least likely to use routine services:

Not time consuming and ideally it doesn’t involve any sort of embarrassing examination or assessment…. especially if you’re trying to get people who don’t see the doctor. [The] breath test or urine test, or something like that would fulfil that requirement. (MV_S05; Delivery stakeholder; Pilot stream)

Another highlighted the uncertainty around threshold-based decisions:

There is a bit of noise in the estimate of the result, such that you’d end up with quite large results where you had a false positive or a false negative as benchmarked against the venous blood draw. (MV_S04; Delivery stakeholder; Pilot stream)

#### RC5: Information, counselling and next-steps pathways

RC5 specified counselling, results communication, next-step information, and safety-netting. Accounts described the mobile encounter as counselling plus testing rather than sample collection alone, particularly when abnormal or uncertain results required explanation and clear onward plans. One delivery stakeholder described counselling as integral:

Lots of men didn’t really know and sort of what to look out for and we would sort of counselled them on that… we would explain that we were offering a PSA blood test and we would explain what the PSA blood test is and what it may mean (MV_S02; Delivery stakeholder; Pilot stream)

One attender described the shock of receiving an abnormal result without adequate preparation:

I was shocked when I got the result [by] the phone call. I was on the phone to my sister at the time and came up and came back. She was the first to say, ‘Oh, don’t worry, that can be negative or a positive thing’. (MV_P15; Attender; Pilot stream)

#### RC6: Data integration and primary care alignment at the mobile-to-usual-care transition

RC6 specified result ownership, record integration, communication confirmation, and alignment with primary care and referral pathways. Accounts distinguished transmission of information from acceptance of responsibility for onward action. One prospective GP stakeholder described the risk of receiving a result without an agreed acceptance point:

There needs to be a carefully managed system, because if we’re just told a result, then we have to refer them.… there might be a lot of over referrals (TPP_GP01; GP stakeholder; Prospective stream)

An attender described fragmentation and the need to navigate gaps personally when ownership for next steps was unclear:

The experience of each of those [prior diagnostic tests] have been dramatically different, as in not as positive in terms of efficiency, having to chase things myself, having to fill the gaps when a referral was proposed that hasn’t taken place. (MV_P08; Attender; Pilot stream)

### Transition-integrity determinant crosswalk and candidate monitoring signals

For transition safeguards, CFIR 2.0 labels were applied descriptively to connect interface risks with iTPP safeguards and candidate monitoring signals for prospective local use. Determinants clustered mainly around available resources, information technology infrastructure, access to knowledge and information, communications, and partnership or ownership boundaries. The crosswalk was supplementary and did not determine register structure, requirement wording, or modality assignment.

**Table 4:** Transition-integrity determinants, specification levers, and candidate monitoring signals

Determinant (domain/construct/subconstruct)	How it produces risk (failure mode)	Specification or governance lever (iTPP anchors)	Candidate signals (examples)
Inner Setting: Available resources	Follow-up depends on discretionary effort. Referrals, repeat tests, or appointments are initiated but not completed, and non-attendance is not systematically tracked or escalated.	The iTPP specifies a minimum operational sequence for abnormal or indeterminate results, including tracking, chasing, and escalation, and maintains named result ownership until acceptance is evidenced (RC6 result ownership; RC5 safety-netting; RC3 downstream workload management).	Proportion of abnormal results with documented next-step action and documented receiving-service acceptance within a defined interval; escalation rate for non-completion.
Inner Setting: Structural Characteristics [Information Technology Infrastructure]	Results and tasks do not reliably enter the receiving record system, so receipt and ownership cannot be verified. Patients can become the de facto courier and safety net.	The iTPP specifies identifier capture and record integration where feasible. Where full integration is unavailable, it specifies an auditable proxy evidence trail (structured result summary plus defined filing or upload process) (RC6 record integration).	Proportion of mobile results filed and coded in the receiving record system within a defined interval; record-matching failure rate.
Outer Setting: Partnerships & Connections	No agreed acceptance point exists between the mobile service, laboratories, primary care, and specialist services. Tasks are pushed between organisations, and workload and liability boundaries remain contested.	The iTPP specifies cross-organisation pathway agreements defining receiving services, acceptance criteria, and escalation routes, including how GP workload is managed and resourced (RC6 primary care alignment; RC3 downstream workload management).	Proportion of abnormal results with documented acceptance by a named receiving service; time from referral initiation to acceptance; referral rejection and rerouting rate.
Inner Setting: Access to Knowledge & Information	Patients and professionals lack durable information about what should happen next, who owns follow-up, and what to do if nothing happens. Delays and dropped handovers are harder to detect.	The iTPP specifies a minimum information set at result communication, including next-step plan, timelines, named responsible service, and escalation instructions. It includes patient-visible confirmation, not only clinician-facing documentation (RC5 next steps; RC6 result ownership).	Proportion of patients receiving a written next-step plan and contact route; proportion of abnormal results with documented follow-up instructions; patient-reported understanding of next steps.
Inner Setting: Communications	Communication routes do not reliably deliver results and instructions, and confirmation of receipt is weak. Failures are discovered late, often through patient chasing.	The iTPP specifies communication channels, redundancy, and confirmation mechanisms, including response expectations and monitoring of failed contacts (RC5 results communication; RC6 closed-loop governance).	Contact success rate and time to successful contact from result availability; proportion with patient-visible confirmation of handover; proportion requiring repeat contact attempts.

Notes. (1) Responsibility acceptance (“loop closure”) is defined as auditable confirmation that a named receiving provider or service has accepted clinical responsibility, not transmission of information alone; (2) Signals are candidate local evaluation and governance measures and do not imply universal thresholds; (3) Determinants are reported using updated CFIR labels at construct level, and at subconstruct level where specified; (4) Traceability of determinant–lever–attribute mapping is provided in Supplementary Table S3 with worked exemplars in Supplementary Appendix A

### Cross-cluster conditionalities and parameter separation

Some requirements were retained in the IF-WHEN layer because applicability depended on operating configuration. Three conditionalities recurred: walk-in models and high-footfall siting increased access but also privacy exposure unless paired with flow controls; faster outreach and booking increased reach but destabilised delivery unless invitation volume was paced to staffing and workflow capacity; and lower-burden modalities or more sensitive thresholds increased participation but also anxiety and downstream workload unless counselling, escalation, safety-netting, and ownership were explicit.

The Evidence and Decision Agenda separated requirements from parameter setting. Numeric thresholds, staffing ratios, invitation pacing, unit costs, funding assumptions, participation gradients, and pathway-completion effects were treated as evidence-needed rather than universal requirements and routed to modelling, economic evaluation, workflow simulation, or local evaluation where not defensibly specifiable from interviews.

## Discussion

### Principal findings

This study developed and demonstrated an implementation-aware Target Product Profile as a method for specifying mobile outreach cancer-testing pathways before rollout. The central finding is practical: a mobile test is not a sufficient implementation object. Stakeholder evidence had to be translated into requirements for invitation, privacy, counselling, documentation, result communication, record integration, referral, and handover before the pathway could be treated as specified.

The Man Van-derived register changed the analytic status of the qualitative material. Concerns about legitimacy, operational capacity, test uncertainty, result ownership, and follow-up did not remain as themes or barriers. They became requirement statements with evidence provenance, consequence-based criticality, conditional triggers, and unresolved parameter fields. This is the method’s main contribution: it separates pathway conditions that can be specified before rollout from parameters that require modelling, evaluation, consensus, or local calibration.

### Interpretation in relation to existing literature

The register’s first contribution is to define the implementation object before strategy selection. Implementation-object and strategy-specification literatures distinguish what is being implemented from the actions used to support adoption, delivery, adaptation, or sustainment [7, 8, 14]. The Man Van-derived register makes that distinction practical. A “mobile test” was too coarse a target for implementation planning because the deliverable pathway also included eligibility explanation, privacy controls, counselling, documentation, result ownership, referral routing, record integration, and loop closure. The register therefore specifies the intervention/pathway object that later strategies would need to support; it is not itself an implementation strategy.

The register’s second contribution is to move adaptation work upstream. Adaptation frameworks help teams record modifications, while core-function/core-form approaches distinguish preserved functions from local forms [26–30]. They do not, on their own, decide which pathway conditions should be treated as candidate minimum safeguards before those forms vary. Recent work on identifying core intervention components in cancer-care scale-up reinforces this gap: fidelity and adaptation frameworks often require teams to decide what is non-negotiable before adaptation, but the practical work of making that decision remains methodologically difficult [38].

The iTPP output supplies that pre-adaptation classification: candidate minimum requirements, conditional triggers, and evidence-needed parameters are made visible before local tailoring. In this sense, iTPP functions as an adaptation-support method at the specification stage. It does not freeze local forms; it states which conditions require preservation, recalibration, or further evidence before forms change.

The MUST layer makes the adaptation claim more precise. Some MUST statements may express core pathway functions, but others are interface safeguards. Privacy protection, result ownership, record integration, and loop closure do not operate like mechanisms of action. They are boundary conditions that keep a mobile pathway from failing when work crosses from outreach into usual care. Calling all of them core functions would over-theorise safeguards; calling none of them candidate minimum requirements would push foreseeable privacy, handover, workload, and safety risks into rollout.

The Evidence and Decision Agenda extends the same argument to parameter setting. Early economic evaluation and diagnostic modelling support explicit treatment of threshold, performance, cost, and capacity assumptions as provisional while evidence is developing [35, 36]. Adaptation, Implementation Mapping, and core-function/core-form literatures similarly show why local forms and parameters should be documented rather than silently standardised [19, 20, 26–30, 35, 36, 39]. The register follows this logic by refusing to fix thresholds, staffing ratios, referral ratios, invitation pacing, pathway-completion effects, and equity effects from interviews alone. It separates what stakeholder evidence can specify as a pathway condition from what modelling, workflow simulation, diagnostic accuracy work, consensus review, micro-costing, or equity-focused evaluation must still estimate. In the Man Van exemplar, contemporaneous point-of-care PSA and micro-costing studies make that separation especially important because test performance, staffing, and resource assumptions remain empirical variables rather than qualitative requirements [40, 41].

Table 2 situates the 42-attribute register among adjacent implementation, design, adaptation, human-factors, and safety approaches. The comparison turns on decision use: iTPP converts stakeholder evidence into consequence-graded, provenance-linked pathway requirements before local adaptation, implementation strategy selection, or prospective evaluation.

**Table 5:** What the Man Van-derived iTPP adds to adjacent approaches at the pre-rollout specification stage

Adjacent approach	What it already supports	What the Man Van-derived iTPP adds
Medical-test Target Product Profiles [9–11]	Intended-use and target-characteristic specification for tests or products, including intended users, performance expectations, and development priorities.	Shifts the specification unit from the test or product to the configured outreach pathway, including invitation, eligibility explanation, privacy, counselling, documentation, result communication, referral, record integration, and handover.
Service-oriented Target Product Profile adaptations [12]	Extension of TPP logic towards service or workflow characteristics.	Adds statement-level criticality, evidence provenance, conditional triggers, and explicit separation of pathway requirements from evidence-needed parameters.
Implementation Mapping and tailored implementation blueprints [19, 20, 39]	Development or tailoring of implementation strategies for adoption, delivery, and sustainment of a defined intervention or pathway.	Specifies the pathway object before strategy selection, making clear what later strategies are intended to help actors adopt, deliver, adapt, or sustain.
Implementation reporting and strategy-specification guidance [8, 17, 18]	More complete description and reporting of interventions, implementation studies, and implementation strategies.	Produces a pre-rollout requirement register before the pathway or strategy package is treated as fully specified.
Adaptation frameworks, including FRAME, FRAME-IS, and ADAPT [26–28]	Documentation or guidance of modifications to interventions and implementation strategies as they move across contexts.	Identifies candidate minimum requirements, conditional requirements, and evidence-needed parameters before local modifications are made.
Core-function/core-form approaches [29, 30]	Distinction between functions that should be preserved and local forms through which those functions are delivered.	Helps distinguish candidate minimum pathway requirements from locally variable forms, while avoiding the assumption that every minimum safeguard is a core function.
Human-centred and user-centred design [21, 22]	Elicitation of user needs, usability problems, contextual fit, and design priorities.	Translates user and stakeholder concerns into pathway requirements with criticality, provenance, and local adaptation conditions.
User-centred design and implementation-science integration [42, 43]	Alignment of usability, workflow, organisational context, implementation feasibility, scale-up, and sustainability during intervention or service development.	Converts workflow and implementation concerns into a pathway specification that can be inspected before local adaptation or strategy selection.
Requirements-development and co-design prioritisation approaches [44, 45]	Development and prioritisation of functional and non-functional requirements, stakeholder values, personas, and evidence-based or experience-based design inputs.	Adds consequence-based pathway criticality, separating candidate minimum safeguards from desirable features, conditional requirements, and unresolved parameters.
Journey mapping and service blueprinting [25]	Mapping of touchpoints, actors, frontstage and backstage processes, and service-interface frictions.	Converts mapped frictions into auditable pathway conditions, including transition-accountability requirements and local adaptation triggers.
SEIPS-informed human factors and community diagnostic service design [23, 46, 47]	Analysis of how people, tasks, tools, environments, organisations, workflows, accessibility, and communication across organisational boundaries shape safe work.	Translates work-system constraints into minimum or conditional pathway requirements and, for transition safeguards, candidate monitoring signals.
Safety-case approaches [24]	Structured argumentation about whether a system is acceptably safe under specified assumptions.	Provides pre-rollout pathway specifications that can feed later assurance work, without substituting for a formal safety case.
Closed-loop result-management and test-result safety literature [4–6]	Evidence that result review, communication, action, follow-up, and loop closure can fail across care interfaces.	Specifies result ownership, record integration, receiving-service acceptance, safety-netting, and loop closure as pathway requirements rather than downstream quality failures.

iTPP = implementation-aware Target Product Profile; SEIPS = Systems Engineering Initiative for Patient Safety. The table distinguishes source roles rather than ranking approaches. The iTPP is presented as complementary to these approaches, not as a replacement for them.

The comparison with service blueprinting is the clearest test of added value. This also protects the method from becoming another barrier-and-facilitator inventory: the register does not simply name constraints, but assigns them to requirement, conditionality, or evidence-needed decision states [48]. Journey mapping and service blueprinting can make touchpoints, backstage work, and service-interface frictions visible [25]. The Man Van-derived register does a different decision job. Walk-in access was not left as an access touchpoint; it became conditional on registration, queue management, staffing, eligibility checks, and privacy controls. Abnormal result communication was not left as a messaging step; it remained incomplete until result ownership, receiving-service acceptance, record integration, safety-netting, and loop closure were specified. The gain from iTPP is therefore not a new qualitative data source. It is a different output form: frictions become consequence-graded pathway requirements, while thresholds, staffing ratios, costs, referral ratios, and equity effects remain evidence-needed parameters.

RC6 shows why access evidence and result-management evidence need to be read together. Mobile cancer-screening reviews explain the access logic of bringing services closer to people [1, 3], while a recent cross-cancer review shows that mobile programmes are also constrained by follow-up coordination, programme cost, equipment and space limits, and referral or reimbursement arrangements [49]. Mobile-clinic cervical-screening evidence similarly highlights the needs of patients with health-related social needs [50]. The Man Van-derived register adds the governance condition: access does not complete the pathway. A result has to be owned, recorded, communicated, accepted by a receiving service when onward action is required, and closed in an auditable way.

The transition attributes convert known result-management failures into pre-rollout specification questions. Mobile mammography and ambulatory test-result studies show failures in follow-up, review, communication, action, and closure [4–6]. Cancer-specific safety-netting guidance treats communication, documentation, follow-up tracking, and inequality-sensitive monitoring as practical safety-netting work [51, 52]. The register turns these failure modes into design questions: who owns the result, where it is recorded, how failed contact or non-completion is detected, who accepts responsibility, and what counts as loop closure. Current UK guidance provides adjacent expectations for acting on test results and managing clinical messages, but not a mobile outreach specification [53–55]. Nankervis et al. add the patient-facing side: blood-test result communication should state what action, if any, is needed next, and electronic access alone is insufficient for all patients or result types [56].

#### Transferability and future research

Transferability lies in the specification procedure rather than in copying the Man Van register. A new pathway would need its own boundary definition, stakeholder evidence, receiving-service agreements, policy constraints, and recalibration of criticality. The transferable test is whether the service can state what must be preserved, what may vary, and which parameters require further evidence before local adaptation.

The following are illustrative transfer cases, not empirical findings about those services. In a mobile colorectal or faecal immunochemical testing pathway, specification would likely emphasise kit distribution, sample return, laboratory processing, threshold-based referral, colonoscopy-capacity routing, and follow-up after abnormal or non-returned tests. Current HPV self-sampling work makes the same point: access depends on eligibility identification, sampling instructions, kit return, laboratory linkage, valid or invalid result handling, incident or harm reporting, and proactive follow-up where digital exclusion, language barriers, or low health literacy affect completion [57, 58]. In community diagnostic services, dominant requirements may concern booking, rooms, result ownership, referral routing, capacity-coupled scheduling, and cross-provider responsibility. Requirement families may recur, but wording, modality, triggers, and evidence-needed parameters depend on cancer type, test modality, population, venue, digital route, policy boundary, and receiving service.

Future work should test procedural transfer. Independent teams should derive attributes and modalities from comparable corpora to assess convergence, divergence, and adjudication needs. Prospective applications should evaluate whether iTPP-informed specifications improve delivery integrity, responsibility transfer, handover documentation, equity monitoring, and adaptation decisions. The Evidence and Decision Agenda should be tested through diagnostic modelling, workflow simulation, micro-costing, budget-impact analysis, consensus review, and equity-focused pathway-completion studies.

#### Implications for implementation, governance, and policy

For implementation teams, commissioners, cancer alliances, and receiving services, the register changes the unit of planning. Teams are not only promoting use of a mobile test; they are specifying the pathway conditions under which the offer remains acceptable, feasible, safe, and evaluable. Invitation channels, eligibility wording, privacy safeguards, counselling expectations, result ownership, record integration, and receiving-service acceptance can be specified as pathway conditions. Diagnostic thresholds, referral ratios, invitation pacing, staffing ratios, unit costs, and equity effects require modelling, costing, workflow analysis, consensus review, or prospective evaluation before they can be treated as stable. The specification should also state who owns the result until loop closure, where the result is recorded, how failed contact is detected, how non-completion is escalated, and when responsibility ends.

The same specification logic matters for English cancer policy, which creates direct-access opportunities without a complete implementation specification. The National Cancer Plan emphasises community diagnostics, digital access, direct access to tests, self-referral, straight-to-test pathways, personalised risk insight, and the NHS App [59]. Where NHS App messages, text messages, emails, or letters are used for invitation or results, the pathway specification should document the approved channel and route owner, information-governance basis, delivery/read-status evidence where available, accessible-format requirements, fallback when App messages are unread or undelivered, and non-digital alternatives for people who cannot or do not use digital channels [60, 61]. Prostate testing illustrates the boundary problem: the UK National Screening Committee does not recommend population prostate screening or targeted screening for any other risk groups, but recommends a tightly bounded targeted programme of two-yearly PSA testing for men aged 45–61 with a pathogenic BRCA2 variant and a relevant family history of breast, ovarian, pancreatic, or prostate cancer, alongside evaluation of how that high-risk group should be identified and invited [62]. Mobile PSA can therefore resemble targeted screening, case-finding, informed access, and community outreach without meeting the authority, invitation rules, or governance requirements of any one category. This ambiguity is an implementation risk: trusted invitation routes, direct access, and community venues can improve reach but make an outreach offer look like screening if rationale, eligibility language, result ownership, and follow-up are underspecified. The iTPP cannot settle screening policy, but it can require specification of who is invited, why, through which channel, with what benefit-harm information, and with what safeguards for communication, referral, record integration, and loop closure. Equity should remain a monitoring issue: the equality impact assessment makes these specification issues concrete by identifying risks around deprivation, ethnicity, disability adjustments, health literacy, digital access and exclusion, trust, GP responsibility boundaries, primary-care access, language access, sex/gender coding for trans women and non-binary people with a prostate, and local mitigation for groups who might otherwise struggle to complete the pathway [63].

#### Strengths and limitations

This study brought together attenders, planning and delivery stakeholders, and a separate Prospective stream; maintained analytic separation between realised and anticipatory evidence; and translated qualitative material into a traceable requirement register. The main strength is auditability: Additional file 2 allows readers to follow the path from evidence stream to attribute, modality, rationale, unresolved parameter, worked exemplar, and worksheet.

Several limitations affect interpretation of the method and register. First, the method was demonstrated by the author team using one empirical exemplar. The accompanying worksheets have not yet been independently applied by external teams, commissioners, service designers, receiving services, or community partners. We therefore cannot claim inter-team consistency, ease of use without expert facilitation, or comparative superiority over intervention-development, design, human-factors, safety-assurance, or implementation-planning approaches. This is a method-development demonstration, not external validation.

Second, the substantive Man Van register should not be treated as a generalisable standard. Some requirement families may recur across outreach pathways, especially legitimacy, informed choice, privacy, counselling or interpretation, result ownership, record integration, and loop closure. However, their wording, modality, triggers, and evidence-needed parameters require local recalibration.

Third, the sample limits equity inference. The Prospective stream was broader and self-selected, so it informed anticipatory requirements rather than observed Man Van performance. Attender interviews were drawn from post-service evaluation respondents who opted into future contact and volunteered to participate, so an invitation response rate cannot be calculated. This recruitment route may have preferentially captured more engaged participants and those with greater digital access. The attender sample also under-represented some groups often prioritised in outreach rationales: most interviewed attenders were White, most reported professional or managerial occupations, no manual workers were identified in the attender sample, and relatively few lived in the most deprived IMD quintile (Table 1). These data do not invalidate requirements concerning legitimacy, privacy, non-digital access, counselling, or handover, but they mean the register cannot be treated as evidence that the pathway reached, worked for, or was equally acceptable to underserved groups.

Fourth, consequence-based criticality involves judgement. Requirement grading could privilege service-system risk if applied only by programme leads or clinical teams. Although patient representatives reviewed the protocol, topic guides, and participant-facing materials, the final register and modality assignments were not subject to a formal patient, community, commissioner, receiving-service, or independent implementation-team consensus process. Future applications should test whether different stakeholder groups assign the same criticality to privacy, digital access, invitation wording, threshold assumptions, result ownership, and handover requirements, and should document disagreement where consensus is not reached.

Finally, this study does not evaluate PSA testing, establish service effectiveness, validate diagnostic thresholds, or provide a recommendation for population screening. The mobile PSA pathway was used as an empirical exemplar for method development. Claims about clinical benefit, screening policy, diagnostic performance, cost-effectiveness, and equity impact require separate evidence.

## Conclusions

Mobile outreach cancer testing should be judged not only by whether testing is brought closer to people, but by whether the pathway can state, before local adaptation, how invitation, privacy, counselling, documentation, result communication, referral, record integration, and responsibility transfer will be governed. The implementation-aware Target Product Profile makes those conditions inspectable by translating stakeholder evidence into auditable requirements, conditional triggers, and evidence-needed parameters. In this mobile PSA exemplar, the method was demonstrated but not externally validated; it did not evaluate service effectiveness, validate diagnostic thresholds, or recommend population screening. Its next test is practical: whether independent teams can use it to preserve safeguards, expose evidence gaps, and identify unsafe local adaptation before rollout.

## Supplementary Information

Below is the link to the electronic supplementary material.

Supplementary Material 1: Additional file 1. DOCX. Completed COREQ checklist for interviews and focus groups.

Supplementary Material 2: Additional file 2. DOCX. Supplementary methodological artefacts for the implementation-aware Target Product Profile: full register (Table S1), Evidence and Decision Agenda (Table S2), determinant crosswalk (Table S3), data dictionary (Table S4), worked translation exemplars (Supplementary Appendix A), and worksheets for replication and local adaptation (Supplementary Appendix B).

## Data Availability

De-identified excerpts and supplementary methodological artefacts supporting the conclusions of this article are included within the article and its additional files. Full transcripts cannot be shared openly because de-identification cannot be fully guaranteed due to the small number of clinical and delivery stakeholders involved.

## Abbreviations

AI: Artificial intelligence
BRCA2: BRCA2 gene
CFIR: Consolidated Framework for Implementation Research
COREQ: Consolidated Criteria for Reporting Qualitative Research
CRUK: Cancer Research UK
GP: General practitioner
HPV: Human papillomavirus
ICB: Integrated care board
iTPP: Implementation-aware Target Product Profile
IMD: Index of Multiple Deprivation
IRAS: Integrated Research Application System
NHS: National Health Service
PSA: Prostate-specific antigen
REC: Research Ethics Committee
SEIPS: Systems Engineering Initiative for Patient Safety
TPP: Target Product Profile
