# Supplementary material for: Specifying pathway requirements for mobile outreach cancer testing before rollout: an implementation-aware Target Product Profile method
Source: Implement Sci Commun. 2026 Jul 16;7:199. doi: 10.1186/s43058-026-01041-7 (PMC13656403; doi:10.1186/s43058-026-01041-7)
Supplement: Supplementary file 1 [file 43058_2026_1041_MOESM1_ESM.docx]

**Additional file 1. Completed COREQ checklist**

*Consolidated Criteria for Reporting Qualitative Research (COREQ): 32-item checklist for interviews and focus groups*

**Manuscript title:** From Evidence to Requirements: The Implementation-Aware Target Product Profile (iTPP) for Specifying Mobile Outreach Cancer Testing Pathways

**How to read this checklist:** The final column gives the page number(s) in the revised manuscript where each item is reported. Items marked “Not reported” are not explicitly stated in the current manuscript text.

| **No.** | **Item** | **Guide questions / description** | **Reported on page no.** |
| --- | --- | --- | --- |
| **Domain 1. Research team and reflexivity** | | | |
| 1 | Interviewer/facilitator | Which author/s conducted the interview or focus group? | p. 6-7 |
| 2 | Credentials | What were the researcher’s credentials (e.g., PhD, MD)? | p. 7 |
| 3 | Occupation | What was their occupation at the time of the study? | p. 7 |
| 4 | Gender | Was the researcher male or female? | p. 6 |
| 5 | Experience and training | What experience or training did the researcher have? | Not explicitly reported |
| 6 | Relationship established | Was a relationship established prior to study commencement? | p. 6-7 |
| 7 | Participant knowledge of the interviewer | What did the participants know about the researcher (e.g., goals, reasons for doing the research)? | p. 6-7 |
| 8 | Interviewer characteristics | What characteristics were reported about the interviewer/facilitator (e.g., bias, assumptions, reasons and interests in the research topic)? | Not explicitly reported |
| **Domain 2. Study design** | | | |
| 9 | Methodological orientation and theory | What methodological orientation underpinned the study (e.g., grounded theory, discourse analysis, ethnography, phenomenology, content analysis)? | p. 4 |
| 10 | Sampling | How were participants selected (e.g., purposive, convenience, consecutive, snowball)? | pp. 4-5 |
| 11 | Method of approach | How were participants approached (e.g., face-to-face, telephone, mail, email)? | pp. 4-6 |
| 12 | Sample size | How many participants were in the study? | pp. 4-6 |
| 13 | Non-participation | How many people refused to participate or dropped out? Reasons? | Not calculable (see limitation statement) |
| 14 | Setting of data collection | Where was the data collected (e.g., home, clinic, workplace)? | p. 6-7 |
| 15 | Presence of non-participants | Was anyone else present besides the participants and researchers? | Not applicable |
| 16 | Description of sample | What are the important characteristics of the sample (e.g., demographic data, date)? | pp. 4-6; Table 1 |
| 17 | Interview guide | Were questions, prompts, guides provided by the authors? Was it pilot tested? | p. 7-8 |
| 18 | Repeat interviews | Were repeat interviews carried out? If yes, how many? | Not applicable |
| 19 | Audio/visual recording | Did the research use audio or visual recording to collect the data? | p. 7-8 |
| 20 | Field notes | Were field notes made during and/or after the interview or focus group? | Not applicable |
| 21 | Duration | What was the duration of the interviews or focus group? | p. 7 |
| 22 | Data saturation | Was data saturation discussed? | p. 4 (specification sufficiency) |
| 23 | Transcripts returned | Were transcripts returned to participants for comment and/or correction? | Not applicable |
| **Domain 3. Analysis and findings** | | | |
| 24 | Number of data coders | How many data coders coded the data? | p. 7 |
| 25 | Description of the coding tree | Did authors provide a description of the coding tree? | pp 7-9 |
| 26 | Derivation of themes | Were themes identified in advance or derived from the data? | pp. 3; 7-9 |
| 27 | Software | What software, if applicable, was used to manage the data? | p. 7 |
| 28 | Participant checking | Did participants provide feedback on the findings? | Not applicable |
| 29 | Quotations presented | Were participant quotations presented to illustrate the themes/findings? Was each quotation identified? | pp. 15-17 |
| 30 | Data and findings consistent | Was there consistency between the data presented and the findings? | pp. 15-17 |
| 31 | Clarity of major themes | Were major themes clearly presented in the findings? | pp. 15-17 |
| 32 | Clarity of minor themes | Is there a description of diverse cases or discussion of minor themes? | pp. 15-17 |
