# Supplementary material for: Specifying pathway requirements for mobile outreach cancer testing before rollout: an implementation-aware Target Product Profile method
Source: Implement Sci Commun. 2026 Jul 16;7:199. doi: 10.1186/s43058-026-01041-7 (PMC13656403; doi:10.1186/s43058-026-01041-7)
Supplement: Supplementary file 2 [file 43058_2026_1041_MOESM2_ESM.docx]

**Supplementary Table S1.** Full attribute-anchored iTPP register with statement-level modalities. MUST/SHOULD/IF‑WHEN reflect the study’s consequence‑based classification, not statutory requirements. Attributes are traceable requirement anchors; modality is assigned to the statement(s) shown within the MUST, SHOULD, and IF-WHEN columns rather than to the attribute label itself.

| iTPP attribute (ID - name) | Minimum (MUST) | Optimal (SHOULD) | Conditional (IF-WHEN) | Evidence provenance |
| --- | --- | --- | --- | --- |
| RC1 – Outreach, invitation and legitimacy | | | | |
| RC1-01  Rationale and eligibility clarity | Provide a clear, plain-language rationale for why the test/service is being offered and who it is intended for. | Include symptom and risk-rationale awareness content (and what to do if symptomatic); do not assume baseline knowledge. | Tailor content to cancer type, literacy level, and local languages; co-produce with community partners where feasible. | Prospective stream |
| RC1-02  Equity-focused targeting and tailoring | Use local intelligence (primary care/public health data plus community knowledge) to prioritise higher-risk and underserved groups and to select or rotate locations accordingly. | Tailor messages, venues and delivery approach to intended groups (for example age/risk, ethnicity, locality), with explicit equity aims stated upfront. | Add additional targeting (for example practice lists) where acceptable, with safeguards to avoid stigma/exclusion and to support those with lower digital access. | Both |
| RC1-03  Legitimacy and trust cues | Provide visible legitimacy and trust signals (NHS/clinical sponsorship, transparent governance, privacy assurances, clear branding) so the service is perceived as safe and credible. | Partner with locally trusted organisations/venues and use consistent, recognisable branding and staff identification to mitigate perceived risk. | Where baseline trust is low, use community champions/peer advocates and/or co-hosted sessions to strengthen legitimacy. | Both |
| RC1-04  Policy- and governance-compatible invitation channel | Provide at least one trusted invitation route compatible with applicable policy and local governance that supports informed choice and does not require a GP appointment (for example, an opt-in, governance-approved GP-endorsed message where permitted, or an equivalent trusted channel), with a clear call-to-action. | Include a one-click/one-scan action link to balanced information and booking (map/slot) within the message, and use reminders where appropriate. | If a consented GP messaging infrastructure exists and local governance permits, use GP-endorsed messaging only for men who have opted in/registered interest (or in response to patient-initiated requests); otherwise use an equivalent trusted messenger/channel. Ensure non-digital alternatives for people without mobile access; avoid digital-only pathways and avoid framing the offer as population screening. | Both |
| RC1-05  Community-embedded venues (workplace, faith, community) | Ensure outreach and/or delivery can occur through community-embedded venues (for example workplaces, community centres, faith settings) to reach people who do not routinely engage through GP appointments. | Select venues based on local routines, footfall and perceived safety; align delivery with existing community events/services to minimise participation burden. | Use pop-ups co-located with complementary services (health checks, community support) when this is expected to improve convenience and perceived legitimacy. | Both |
| RC1-06  On-site discovery cues (signage/wayfinding) | Provide clear on-site discovery cues (visible signage/wayfinding at the host venue) so eligible individuals can identify the service confidently. | Use multiple discovery triggers (signage plus staff prompts plus partner signposting) to support opportunistic engagement without confusion. | Where venue norms require discretion, design prompts to be visible but not stigmatising (for example neutral health-check framing). | Pilot stream |
| RC1-07  Opportunistic walk-up engagement | Make an explicit design decision about walk-in versus booked access, and align staffing/space accordingly to avoid unmanaged queues and visible waiting. | In high-footfall locations, enable opportunistic walk-in capture with a simple front-door triage and clear time expectations. | Where privacy or throughput is constrained, restrict walk-ins (or allocate limited walk-in slots) and communicate this clearly. | Both |
| RC1-08  Printed outreach materials (posters/leaflets) | Provide concise printed materials (leaflets/posters) explaining purpose, eligibility, what will happen, privacy, and how results/next steps work. | Use visually clear, low-literacy formats with community-tested wording and translations where needed. | Combine printed materials with QR codes or short links to richer information for those who prefer digital detail. | Both |
| RC1-09  Multi-channel outreach (avoid GP-text only) | Avoid single-channel outreach; plan for at least one non-GP route to reach people who do not respond to GP text invitations. | Implement a tailored channel-mix strategy (GP texts plus community venues plus local prompts plus partner signposting), adapting by locality and target group. | Use rapid monitoring of reach (who is and is not attending) to adjust the channel mix in-flight. | Pilot stream |
| RC1-10  Engagement assets (FAQ/scripts/community champions) | Provide core engagement assets (brief FAQ/script) that support accurate, consistent messaging and informed participation across outreach channels. | Include a feedback mechanism to iteratively refine outreach assets and messaging where engagement is low, subject to governance and resource capacity. | If mistrust is anticipated, use locally trusted messengers and/or co-designed materials, and include explicit explanations of data use, confidentiality and what happens next. | Pilot stream |
| RC2 – Minimising friction while protecting privacy and dignity | | | | |
| RC2-01  Access model and hours (fit with daily life) | Configure access so attending does not require substantial time off work or complex travel; offer appointment times that fit local working patterns. | Provide out-of-hours/weekend availability and/or rotating site times to match local working routines. | If appointment slots are limited, prioritise equity-relevant groups and provide clear alternatives to avoid exclusion. | Both |
| RC2-02  Booking workflow (QR/online self-booking) | Provide a clear booking route that does not depend on digital access (for example phone or assisted booking) and that supports basic eligibility checks. | Provide QR/online self-booking with appointment slots, automated confirmations/reminders, and clear pre-visit information. | Offer assisted digital support (at the venue or via partner organisations) to mitigate digital exclusion. | Both |
| RC2-03  Access model (appointment vs walk-in) | Specify whether the service is appointment-based, walk-in, or mixed, and provide basic queue/wait management to protect privacy and throughput. | Use a mixed model (booked backbone plus managed walk-in capacity) calibrated to venue footfall and staffing. | Use timed tickets/SMS queue updates where waiting visibility is a concern or where space is limited. | Both |
| RC2-04  On-site experience (comfort and perceived quality) | Ensure the on-site environment is professional, clean and respectful, so the mobile offer is perceived as legitimate healthcare (not a pop-up gimmick). | Maintain a high-quality set-up (clear wayfinding, respectful interpersonal approach, consistent staff presence) to reinforce trust and comfort. | Where feasible, provide a private consultation space separate from waiting/entry points. | Both |
| RC2-05  Waiting, privacy and visibility | Protect privacy for conversations and sample collection, and manage waiting so people are not visibly exposed or embarrassed. | Use physical screens, discrete entry/exit, separate waiting zones, and/or scheduling to mitigate concerns about visible waiting and perceived exposure. | If privacy cannot be assured on-site for a given sample type, offer an alternative (for example home collection or different modality) rather than forcing an uncomfortable process. | Both |
| RC2-06 - Travel/time burden and convenience | Site delivery to minimise travel time and cost for intended groups (including convenient public transport access where relevant). | Locate services in familiar, locally safe venues and/or rotate sites to minimise repeated travel burdens. | Where travel is unavoidable, consider travel support or scheduling that minimises opportunity cost (for example co-location with other appointments). | Both |
| RC3 – Deliverability, staffing, and sustainability | | | | |
| RC3-01  Funding model and sustainability | Specify how the service could be sustained beyond a one-off pilot, including funding and governance arrangements. | Develop a sustainability model that explicitly accounts for staffing, consumables, coordination overhead and follow-up pathway costs. | Use phased rollout (pilot to targeted expansion to routine service) with explicit go/no-go criteria tied to deliverability and equity impact. | Both |
| RC3-02  Downstream workload management | Define who owns results and who is responsible for follow-up; do not default to unplanned GP workload without resourcing and explicit agreements. | Implement defined routing to appropriate pathways (for example specialist referral hubs) and use central coordination to prevent follow-up fragmentation. | Provide GP notification for continuity without transferring responsibility unless formally agreed and resourced. | Prospective stream |
| RC3-03  Operational intensity and staffing burden | Design the delivery workflow to be feasible with realistic staffing; avoid labour-intensive steps as the default operating mode. | Streamline on-site tasks via pre-registration, standardised scripts, and clear role allocation to minimise operational friction. | Use trained volunteers/community staff for non-clinical roles where appropriate, with clear boundaries and supervision. | Both |
| RC3-04  Partnership and coordination overhead | Establish a partnership model (integrated care board, public health, clinical provider, and community host) with clear roles, responsibilities and escalation routes. | Plan coordination tasks explicitly (venue permissions, community engagement, data governance, communications) to minimise last-minute friction. | Use a single coordinating function (or delivery hub) to consolidate operational tasks across partners where system structure allows. | Both |
| RC3-05  Staffing cost and grade | Specify required staff roles and associated costs (including clinical oversight needs) as part of the iTPP deliverability specification. | Enable delivery by appropriately trained non-doctor staff (for example nurses/HCAs) where clinically safe, reserving GP input for escalation/complex counselling. | Where clinical risk is higher (for example complex eligibility or results), define additional clinical oversight requirements explicitly. | Prospective stream |
| RC3-06  Skill-mix and task-shifting | Define the required skill mix, training and competency pathways (including escalation protocols) to support safe task-shifting. | Use competency-based sign-off and refresher training to maintain fidelity as staff rotate and as test modalities evolve. | Where staff turnover is high, build in rapid onboarding materials and supervision to preserve delivery quality. | Both |
| RC4 – Test modality acceptability and performance thresholds | | | | |
| RC4-01  Acceptability: non-invasive testing and invasive follow-up | Treat acceptability as dependent on downstream consequences; specify referral criteria and follow-up pathways to minimise unnecessary invasive procedures and avoid avoidable harm. | Provide balanced pre-test information about the possibility and nature of follow-up testing so individuals can make an informed choice. | Where follow-up is likely to be invasive or delayed, strengthen counselling and consider alternative testing offers/thresholds to mitigate potential harms. | Both |
| RC4-02  Breath testing feasibility and privacy | Select sample modalities that are feasible in a mobile setting without compromising privacy, infection control or throughput. | Where alternative modalities are considered, prioritise lower-burden modalities (for example breath testing, finger-prick blood) where appropriate; ensure adequate privacy space if urine collection is required. | Where privacy space is limited, prefer modalities that do not require intimate facilities (for example breath/saliva/blood) rather than forcing uncomfortable collection. | Prospective stream |
| RC4-03  Procedure clarity (avoid feared procedures) | Provide a clear explanation of the on-site process (what happens in the van) to address anticipatory anxiety and procedural misconceptions. | Use pre-visit materials and staff scripts to clarify what will and will not happen (including what procedures are not part of the visit). | Where misunderstanding persists, incorporate brief walk-through descriptions, visuals, or peer reassurance to address fear-based barriers to attendance. | Pilot stream |
| RC4-04  Minimising harm from confirmatory investigations | Build harm minimisation into design (avoid false reassurance or unnecessary alarm; ensure timely routing of abnormal results). | Communicate false-positive/false-negative risk and the possibility of downstream invasive testing in balanced terms; provide supportive counselling and clear next steps. | For tests with higher uncertainty, strengthen safety-netting and plan additional support contacts to mitigate avoidable distress. | Pilot stream |
| RC4-05  Performance thresholds (evidence-needed field) | Define intended use, state the provisional performance hypothesis, and mark numeric thresholds as evidence-needed until supported by diagnostic accuracy evidence, modelling, or consensus review. | Align performance thresholds with clinical consensus, pathway capacity and equity implications; revise iteratively as evidence matures. | Treat numeric performance thresholds as an evidence-needed field in early iTPP versions; avoid hard numeric targets unless supported by validated studies. | Prospective stream |
| RC4-06  Sample type preferences (breath/urine/blood vs stool) | Prioritise low-burden, non-invasive sample types suitable for mobile delivery (breath/saliva/urine/finger-prick blood) and provide required facilities/equipment. | Offer the least intrusive feasible modality for the intended use-case, balancing acceptability with operational feasibility. | Use stool-based sampling only when required by the test; consider home collection and return logistics when stool is unavoidable. | Both |
| RC4-07  Accuracy, false positives and acceptability | Be explicit about test limitations and threshold choices (false positives/negatives); avoid implicit performance claims in outreach and consent materials. | Use evidence-based thresholds and communicate accuracy in accessible language that supports informed participation. | Update thresholds and communications as evidence evolves; maintain version control for the iTPP to prevent message drift. | Both |
| RC4-08  Acceptable trade-offs (false positives vs missed cases) | Make explicit which trade-offs are being made (accuracy vs accessibility vs capacity vs equity) and who has decision authority. | Incorporate stakeholder preferences and equity impact assessment when setting trade-offs; document rationale in the iTPP version history. | Where trade-offs are contested, run structured deliberation/consensus steps (and document dissent) rather than forcing false agreement. | Prospective stream |
| RC5 – Information, counselling and next-steps pathways | | | | |
| RC5-01  Counselling and interpretation capability | Ensure access to counselling/interpretation for results with significant implications (on-site or tightly linked), so uncertainty and next steps can be managed safely. | Provide trained staff and clear materials to support informed consent, result interpretation and referral discussions in the mobile context. | Use remote counselling/telehealth support where on-site capacity is limited, with clear escalation pathways. | Both |
| RC5-02  Expectation-setting: test limitations | Set expectations that the test is not definitive and that uncertainty is possible; avoid framing that implies a guaranteed all-clear. | Use consistent, balanced benefit/harms messaging across staff and materials to prevent overselling and to support informed choice. | Where anxiety risk is higher (for example cancer-fear contexts), incorporate additional reassurance about support and next steps rather than over-promising certainty. | Prospective stream |
| RC5-03  Standardised scripts and guidance | Provide structured scripts/guidance to standardise core information (eligibility, what happens, limitations, next steps) and minimise message drift. | Train staff in script use and update guidance as test modalities and pathways change; audit fidelity where feasible. | Where multiple partners deliver, agree a shared script and governance process to avoid contradictory messaging. | Prospective stream |
| RC5-04  Next steps and timelines | Provide a clear description of what happens after testing (results pathway, referral options, expected timelines, and contact routes). | Provide a simple pathway map (written/digital) that individuals can take away, including who to contact if results are delayed or symptoms change. | Tailor next-step information by risk level/result category (for example normal vs borderline vs abnormal) to mitigate confusion and anxiety. | Prospective stream |
| RC5-05  Safety-netting and escalation | Provide safety-netting instructions and escalation routes for concerning symptoms or delays, independent of the mobile visit. | Implement proactive follow-up/track-and-trace for abnormal or unclear results to prevent people falling through gaps. | Where pathway capacity is constrained, define interim support steps (for example symptom monitoring guidance or rapid re-contact options). | Both |
| RC5-06  Result timing and communication to address anxiety/harm concerns | Communicate expected result timeframes upfront to support expectation-setting and reduce uncertainty-driven unplanned help-seeking. | Offer patient-centred result timing/communication options (within clinical constraints) to address anxiety and disruption concerns. | Where rapid results are not possible, provide interim check-ins or clear when-to-worry guidance to mitigate distress. | Pilot stream |
| RC5-07  Results communication (timing and method) | Define how results will be communicated (method, timeframe) and ensure confidentiality and accessibility. | Offer multiple communication modes (SMS/phone/letter/portal) depending on result sensitivity and patient preference. | For abnormal results, prioritise clinician-led contact with clear referral instructions; for normal results, low-burden automated routes may be acceptable. | Both |
| RC6 – Data integration and primary care alignment | | | | |
| RC6-01  Data and record integration | Ensure encounter data and results are captured and accessible for ongoing care (for example can be communicated to the GP record) with appropriate data governance, including documentation of result ownership and (where onward action is required) whether loop closure has occurred. | Integrate electronically with NHS/primary care systems to minimise manual transfer and information loss. | If full integration is not feasible, provide a structured patient‑held result summary and a defined process for uploading to clinical records, including how result ownership and loop‑closure status will be documented. | Both |
| RC6-02  Primary care access friction | Design follow-up assumptions acknowledging that access to GP appointments may be constrained; do not assume rapid GP follow-up as the default route. | Provide follow-up routes that bypass GP bottlenecks where appropriate (for example direct referral hubs or specialist advice lines) while keeping primary care informed. | Provide navigation support (who to contact, how to escalate) for individuals who struggle to access routine services. | Both |
| RC6-03  Result ownership and GP workload | Specify ownership and responsibility for acting on results within the mobile‑testing pathway until loop closure is confirmed, where loop closure is defined as positive confirmation of transfer of clinical responsibility to a named receiving provider/service/team, evidenced in an auditable way (not merely the transmission of information). Do not transfer workload to GPs without explicit agreement and resourcing. | Use central coordination and clear handoffs to secondary care/specialist pathways to mitigate fragmentation and duplicated work, including explicit identification of the receiving provider/service/team and the confirmation route used to evidence loop closure. | Where responsibility is intended to transfer to primary care, specify the acceptance/acknowledgement mechanism and resourcing that evidences loop closure; provide concise GP summaries for continuity without implying transfer of responsibility unless explicitly agreed and acknowledged. | Both |
| RC6-04  Alignment with screening/referral pathways | Map how mobile testing outcomes connect to existing screening/referral guidance and local services to prevent fragmentation. | Align criteria and communications with NHS pathways (and local referral processes) so the mobile offer feels like an extension of usual care. | Where local pathways differ, document localisation explicitly in the iTPP (site-specific implementation annex). | Prospective stream |
| RC6-05  Primary care involvement and pathway leadership | Maintain trust by ensuring primary care is informed and engaged, even when testing occurs outside GP premises. | Co-design communications with GPs and define pathway leadership/accountability so patients perceive continuity rather than a detour from care. | Where GP involvement is limited, provide alternative trusted points of contact (for example specialist nurse/helpline) to sustain credibility. | Prospective stream |

**Supplementary Table S2. Evidence and Decision Agenda (parameters excluded from the iTPP to avoid false precision).**

| Workstream | Parameter Category | Quantitative/Operational Goal | Methodological Requirement |
| --- | --- | --- | --- |
| 1. Clinical Performance | Diagnostic Thresholds | Define optimal PSA/biomarker cut-points for community triage. | Diagnostic accuracy modelling and consensus review. |
|  | Referral Ratios | Quantify expected false-positive/negative rates in low-prevalence settings. | Longitudinal pathway tracking and yield analysis. |
| 2. Operational Capacity | Staffing Ratios | Determine specific staff-to-patient ratios required to manage throughput. | Workflow simulation and time-motion studies. |
|  | Outreach Pacing | Define the maximum rate of invitations/appointments that can be safely handled given staffing levels, workflow steps, and available space (to avoid unmanaged queues and unsafe throughput). | Capacity-demand modelling of booking systems. |
| 3. Health Economics | Unit Costs | Establish per-test and per-referral costs for the mobile delivery model. | Micro-costing and early economic evaluation. |
|  | Service funding and price points | Define the "blended" payment rate to fund tracking and results ownership. | Budget impact analysis across primary/secondary care interfaces. |
| 4. Equity & Access | Participation Gradients | Measure the impact of iTPP requirements on attendance from underserved groups. | Geospatial utilization analysis and comparative cohort studies. |
|  | Pathway Completion | Test whether MUST-level interface controls reduce time-to-resolution. | Comparative analysis of mobile vs. fixed-site diagnostic timelines. |

**Supplementary Table S3. Crosswalk linking Table 3 safeguards and Table 4 determinants to specific iTPP attribute IDs (Table S1).**

| Source | Source Row ID | Tension or Determinant Label | Safeguard / failure mode | Linked attribute ID(s) | Linked attribute name(s) |
| --- | --- | --- | --- | --- | --- |
| Table 3 | T3-01 | Trust outside clinical buildings | Verifiable clinical sponsorship and identifiable governance sponsor; authenticated invitation route plus non-digital access route; clear rationale and eligibility messaging (RC1). | RC1-03; RC1-04; RC1-09; RC1-01 | Legitimacy and trust cues Policy- and governance-compatible invitation channel; Multi-channel outreach (avoid GP-text only); Rationale and eligibility clarity |
| Table 3 | T3-02 | Visibility versus privacy in community venues | Privacy-by-design layout and workflow controls; managed waiting and discrete flow; explicit access model that avoids unmanaged queues (RC2, linked to RC1 discovery cues). | RC2-05; RC2-03; RC2-04; RC1-06; RC1-07 | Waiting, privacy and visibility; Access model (appointment vs walk-in); On-site experience (comfort and perceived quality); On-site discovery cues (signage/wayfinding); Opportunistic walk-up engagement |
| Table 3 | T3-03 | Demand versus on-unit capacity | Capacity-coupled outreach and booking; workflow deliverability specified against realistic staffing; explicit walk-in policy aligned to prerequisites (RC3, with RC5 counselling implications). | RC3-03; RC2-02; RC1-07; RC5-01 | Operational intensity and staffing burden; Booking workflow (QR/online self-booking); Opportunistic walk-up engagement; Counselling and interpretation capability |
| Table 4 | T4-01 | Inner Setting: Available resources | Follow-up depends on discretionary effort; referrals/repeat tests initiated but not completed; non-attendance not tracked/escalated. | RC6-03; RC5-05; RC3-02 | Result ownership and GP workload; Safety-netting and escalation; Downstream workload management |
| Table 4 | T4-02 | Inner Setting: Structural Characteristics [Information Technology Infrastructure] | Results/tasks do not reliably enter receiving record; receipt/ownership cannot be verified; patients become de facto courier. | RC6-01 | Data and record integration |
| Table 4 | T4-03 | Outer Setting: Partnerships & Connections | No agreed acceptance point between mobile service/labs/primary care/specialists; tasks pushed between organisations; liability boundaries contested. | RC6-03; RC3-02; RC3-04 | Result ownership and GP workload; Downstream workload management; Partnership and coordination overhead |
| Table 4 | T4-04 | Inner Setting: Access to Knowledge & Information | Patients/professionals lack durable info on next steps, ownership, what to do if nothing happens; delays harder to detect. | RC5-04; RC5-03; RC5-01; RC6-03 | Next steps and timelines; Standardised scripts and guidance; Counselling and interpretation capability; Result ownership and GP workload |
| Table 4 | T4-05 | Inner Setting: Communications | Communication routes do not reliably deliver results/instructions; confirmation of receipt weak; failures discovered late via patient chasing. | RC5-07; RC5-06; RC5-05; RC6-03 | Results communication (timing and method); Result timing and communication to address anxiety/harm concerns; Safety-netting and escalation; Result ownership and GP workload |

**Supplementary Table S4. Data Dictionary.**

| Dataset | Field | Definition | Type/Format | Notes |
| --- | --- | --- | --- | --- |
| Supplementary Table S1 (iTPP register) | Cluster_ID | Requirement cluster identifier (RC1–RC6). | text |  |
| Supplementary Table S1 (iTPP register) | Cluster_name | Requirement cluster name. | text |  |
| Supplementary Table S1 (iTPP register) | Attribute_ID | Unique attribute identifier (RC#-##). | text |  |
| Supplementary Table S1 (iTPP register) | Attribute_name | Short attribute label. | text |  |
| Supplementary Table S1 (iTPP register) | Minimum_MUST | Minimum (MUST) requirement statement (consequence-based criticality; not legal obligation). | text |  |
| Supplementary Table S1 (iTPP register) | Optimal_SHOULD | Optimal (SHOULD) requirement statement (desirable optimisation). | text |  |
| Supplementary Table S1 (iTPP register) | Conditional_IF_WHEN | Conditional (IF-WHEN) requirement statement triggered by configuration/context. | text |  |
| Supplementary Table S1 (iTPP register) | Evidence_base | Evidence provenance notes / trace hooks to supporting excerpts or stream provenance (as provided in the register). | text |  |
| Supplementary Table S2 (Evidence and Decision Agenda) | Workstream | Workstream grouping for parameterisation/evidence needs. | text |  |
| Supplementary Table S2 (Evidence and Decision Agenda) | Parameter Category | Parameter category requiring local calibration or additional evidence. | text |  |
| Supplementary Table S2 (Evidence and Decision Agenda) | Quantitative/Operational Goal | Decision-relevant quantitative/operational question to be addressed. | text |  |
| Supplementary Table S2 (Evidence and Decision Agenda) | Methodological Requirement | Indicative study/evidence approach needed to address the parameter question. | text |  |
| Supplementary Table S3 | Source | Manuscript artefact where the mapped tension/determinant is summarised (e.g., Table 3, Table 4). | Text | Text label; used for traceability. |
| Supplementary Table S3 | Source Row ID | Row identifier within the source artefact. | Text | Formatted as T3-## etc. |
| Supplementary Table S3 | Tension or Determinant Label | Label for the cross-cutting tension or determinant signal being mapped. | Text |  |
| Supplementary Table S3 | Safeguard / failure mode | Description of the failure mode and/or corresponding safeguard encoded in the iTPP. | Text |  |
| Supplementary Table S3 | Linked attribute ID(s) | iTPP attribute identifier(s) linked to the tension/determinant. | Text | Semicolon-separated list of RC#-## codes. |
| Supplementary Table S3 | Linked attribute name(s) | Attribute name(s) corresponding to the linked iTPP attribute IDs. | Text | Semicolon-separated list aligned to Linked attribute ID(s). |
| Supplementary Appendix A | Source (group; stream) | De-identified source label indicating participant group and evidence stream for each worked exemplar. | Text | Not a unique trace ID. |
| Supplementary Appendix A | iTPP attribute (ID name) | iTPP attribute ID and short name produced from the exemplar. | Text | Attribute ID formatted as RC#-##. |
| Supplementary Appendix A | Modality / provenance | Criticality modality (Minimum/Optimal/Conditional; MUST/SHOULD/IF-WHEN) and evidence provenance (pilot/prospective/both). | Text | Criticality is consequence-based, not legal obligation. |
| Supplementary Appendix A | Verbatim excerpt (de-identified) | De-identified verbatim transcript excerpt supporting the exemplar. | Text | Identifiers removed. |
| Supplementary Appendix A | Framework label(s) | Analytic label(s) applied to the excerpt (e.g., determinant taxonomy labels where relevant). | Text |  |
| Supplementary Appendix A | Consequence-based modality rationale | Rationale for the modality assignment based on plausible harm or predictable failure if unspecified. | Text |  |
| Supplementary Appendix B | Worksheet_ID | Identifier for worksheet B1–B6. | Text | Local users may add or remove worksheets. |
| Supplementary Appendix B | Field | Worksheet field name. | Text | Prompt field, not a validated measurement variable. |
| Supplementary Appendix B | Prompt | Question or instruction guiding local application. | Text | Intended for replication and local testing. |
| Supplementary Appendix B | Local response | User-entered response during local application. | Free text | Not part of the present study dataset. |
| Supplementary Appendix B | Evidence/provenance | Source used to support the local response. | Text | May include interview evidence, policy, audit data, stakeholder input, or local records. |

**Supplementary Appendix A. Worked translation exemplars (n=11) linking illustrative quotations to framework charting, iTPP attribute translation, and modality rationale. Worked traces illustrate statement-level modality assignment within named iTPP attributes.**

| Source (group; stream) | iTPP attribute (ID name) | Statement-level modality / provenance | Illustrative quote(s) | Framework label(s) | Consequence-based modality rationale |
| --- | --- | --- | --- | --- | --- |
| Attender; Pilot Stream | RC1-03  Legitimacy and trust cues | MUST; Both | “Getting a text message from the surgery helped, because that is I get regular messages from them. So, it's useful to know it's coming from the same source and the source I recognize….”  “The branding of the Van helped… Having prostate cancer written all over it helps jog people's memory and putting a little leaflet box outside. So, if I'm walking by as going into IKEA, I go “Oh, that's interesting. I'll take a leaflet to see what I know. How do I get onto the bandwagon to get tested?” | Trust/legitimacy cues (GP-endorsed invitation + recognisable branding); Recognisable NHS/GP provenance reduces scam concerns and supports uptake | If legitimacy cues are weak, invitations are screened as scams and eligible individuals do not attend; reach becomes skewed. |
| Man Van planning/delivery stakeholders; Pilot Stream; | RC1-07  Opportunistic walk-up engagement | IF-WHEN; Both | “We wanted it to be a walk in service, initially, but it doesn't work because people had to be registered pre-registered. So, I suppose having the opportunity to have it as a walk in service would be great. I guess in terms of spacing, it would be good if you could do sort of not as a one-stop shop but do sort of those other investigations like blood tests and everything.” | Access model feasibility (walk-in vs preregistration); Walk-in access constrained by registration/eligibility prerequisites | If walk-in access is assumed without prerequisites and capacity controls, unmanaged demand and admin constraints disrupt delivery and create privacy/throughput failures. |
| Attender; Pilot Stream | RC1-04  Policy- and governance-compatible invitation channel | MUST; Both | “I do tend to get texts from time to time from the GP. I didn't I didn't think it was a scam. I probably googled it or asked around.” | Trusted invitation route (GP-endorsed SMS) reduces perceived scam risk; Routine GP-text familiarity legitimises the offer | If invitation routes are not trusted, individuals may ignore/avoid the service, limiting reach and worsening equity. |
| Man Van planning/delivery stakeholders; Pilot Stream | RC2-05  Waiting, privacy and visibility | MUST; Both | “We had a tiny little waiting room with a folding door but unfortunately the soundproofing wasn't very good so we could if there was a second patient came he would have to wait in the waiting area in maybe it was a doctor surgery or wherever” | Privacy-by-design waiting/conversation controls (acoustics + managed waiting); Poor soundproofing + shared waiting creates confidentiality risk | If privacy is not protected, conversations can be overheard; disclosure and acceptability drop; confidentiality breaches occur. |
| Man Van planning/delivery stakeholders; Pilot Stream | RC2-02  Booking workflow (QR/online self-booking) | SHOULD; Both | “We did have leaflets to hand out which had the QR code on that they could scan and book an appointment and obviously we would tell them exactly what we would do when they came to their appointment.… They came to the Van, we were able to explain a bit more in detail.” | QR/online booking supports low-friction access and slot control; QR-coded leaflets used to support scanning and booking | If digital booking is absent (where appropriate), friction increases and staff time is consumed by manual scheduling; throughput suffers. |
| Man Van planning/delivery stakeholders; Pilot Stream | RC3-03  Operational intensity and staffing burden | MUST; Both | “Once we started sending text to the GP surgery, you got a pretty reliable take up rate per output of text. So that allowed us to quite precisely tailor the workflow coming through what somebody's got to face to, to the to the available manpower, staffing, whatever in the van. So, we were able to go from 20 patients a week to sort of 20 patients a day or give or take 100 patients a week, which was pretty good, given the amount of healthcare testing that we were doing per patient.” | Capacity-coupled outreach and staffing feasibility (invitation pacing to manpower); Outreach volume must be paced to available staff/workflow to avoid unsafe compression | If outreach volume outpaces staffing, counselling/documentation is compressed, queues become visible, and errors increase. |
| Man Van planning/delivery stakeholders; Pilot Stream | RC5-01  Counselling and interpretation capability | MUST; Both | “We would counsel about prostate cancer and the risks of prostate cancer. So, identifying if they had any family history, [and] sort of explaining about prostate cancer. Lots of men didn't really know what to look out for and we would sort of counsel them on that there isn't always something to look out for. We would explain that we were offering a PSA blood test and we would explain what the PSA blood test is, and what it may mean what the next steps would look like. If the PSA was raised, we would also check the urine of men as well. So, if they had sort of severe lower urinary tract symptoms, we would explain to them what that may mean, and what next steps might look like. But we would also reassure them that sometimes it's absolutely nothing.” | Counselling and interpretation are integral to mobile testing encounter; Men need explanation of PSA test and what results/next steps mean | If counselling/interpretation is absent, informed choice is undermined and abnormal results can cause avoidable distress or poor follow-up. |
| Man Van planning/delivery stakeholders; Pilot Stream | RC5-05  Safety-netting and escalation | MUST; Both | “Once they had the appointment, we gave them our number, and they could always ring us… I think with the tests that went on, they were grateful when they got their phone call. We told them their phone call would be the following week, and they would get the results of the blood tests and the urine test”. | Active safety-netting via staffed contact route after visit; Participants use helpline/number to understand next steps and reduce drop-off | If safety-netting is absent, uncertainty and missed contacts can lead to people falling through gaps after abnormal/unclear results. |
| Stakeholder–GP; Prospective Stream | RC6-03  Result ownership and GP workload | MUST; Both | “There needs to be a carefully managed system because if we're just told a result, then we have to refer them… There might be a lot of over-referrals as well as appointment time. I think the mobile clinic team should be responsible for the result and referring. Ideally, there'd be a direct referral from there to the hospitals, and the GP is just informed that the correct management's taken place”. | Defined ownership/acceptance point prevents unplanned GP workload transfer; 'Results sent' ≠ 'responsibility accepted'; unmanaged transfer risks over-referral | If ownership and acceptance are unclear, responsibility is pushed to GPs by default, increasing liability and potentially generating inappropriate referrals. |
| Attender; Pilot Stream | RC6-01  Data and record integration | MUST; Both | “What I mean by joined up is and I gave blood to the Man Van service once and the results came back via my GP after a number of weeks. The question is, how do you then join that up with my medical data which has been assembled about me from when we came to this country, with lots of records, etc. Over the years, you've gone from handwritten records to automating the collection of data to maybe in the cloud… So, if you had the data sort of organized in a reasonable way, then you could ‘mine’ it...” | Need to 'join up' data/records across fragmented NHS systems; Disparate processes and records motivate integration for continuity | If encounter data/results are not captured into a durable record, continuity is lost and responsibility transfer cannot be audited. |
| Attender; Pilot Stream | RC5-06  Result timing and communication to address anxiety/harm concerns | MUST; Pilot | “I was shocked when I got the result [by] the phone call. I was on the phone to my sister at the time and came up and came back. She was the first to say, ‘Oh, don't worry, that can be negative or a positive thing’” | Result communication can trigger anxiety; timing/mode needs expectation-setting; Unexpected abnormal results by phone can cause shock without preparation | If result timing/mode is not expectation-set, participants may experience avoidable distress and seek unplanned care; follow-up may be destabilised |

**Supplementary Appendix B. Worksheets for applying the implementation-aware Target Product Profile method**

These worksheets are intended to support replication, teaching, and local testing of the implementation-aware Target Product Profile method. They are guidance materials, not a validated instrument. Users should adapt them to the test, pathway, setting, governance arrangements, and receiving services under consideration. The worksheets are designed to help users define the pathway boundary, chart stakeholder evidence, translate evidence into requirement statements, assign consequence-based criticality, separate evidence-needed parameters, and record local adaptation decisions.

The field names in Worksheets B1–B6 are prompts rather than analytic variables. They are intended to support local application and should be adapted where local governance, policy, or service configuration requires different fields.

**Worksheet B1. Pathway boundary and intended use**

| **Field** | **Prompt** |
| --- | --- |
| Test, service, or pathway being specified | What test, service, or pathway is being specified? |
| Intended use | Is the pathway intended for screening, targeted screening, triage, case-finding, risk assessment, symptom assessment, follow-up, or another use? |
| Intended population | Who is intended to receive the offer? |
| Pathway start point | Where does the pathway begin: invitation, referral, self-referral, walk-up, booking, or another point? |
| Pathway end point | Where does the mobile or outreach pathway end: result communication, referral, receiving-service acceptance, diagnostic resolution, or another point? |
| Delivery setting | Mobile unit, community venue, workplace, home, primary care, community diagnostic centre, digital pathway, or other setting. |
| Receiving services | Which service, team, or provider receives responsibility after the outreach encounter? |
| Excluded claims | What does this specification not claim, for example effectiveness, recommendation for population screening, validated thresholds, or universal transferability? |
| Policy or guidance boundaries | What national or local guidance constrains invitation, eligibility, consent, referral, screening language, or result ownership? |
| Local adaptation boundary | Which elements are expected to remain stable, and which may vary locally? |

**Worksheet B2. Evidence charting**

| **Field** | **Prompt** |
| --- | --- |
| Source ID | Participant group, document, observation, stakeholder source, or local evidence source. |
| Evidence excerpt or summary | What was said, observed, reported, or documented? |
| Evidence stream or provenance | Pilot, Prospective, both, local stakeholder evidence, policy/guidance source, audit data, or other source. |
| Actor | Who acts or is expected to act? |
| Pathway step | Invitation, booking, attendance, testing, counselling, result communication, referral, handover, follow-up, or evaluation. |
| Setting or context | Mobile unit, community venue, workplace, home, primary care, specialist service, laboratory, digital system, or community diagnostic service. |
| Failure risk | What could go wrong if this issue is not specified? |
| Affected group | Which patients, staff, services, communities, or system partners could be affected? |
| Candidate requirement idea | What requirement might follow from this evidence? |
| Uncertainty | What remains unclear or contested? |

**Worksheet B3. Requirement translation and criticality assignment**

| **Field** | **Prompt** |
| --- | --- |
| Attribute ID | Use RC#-## or a local equivalent. |
| Attribute name | Short label for the requirement anchor. |
| Requirement statement | Write as a delivery or handover condition, not as a theme. |
| Criticality | MUST, SHOULD, or IF-WHEN. |
| Criticality rationale | What harm, predictable delivery failure, unsafe handover, or inequitable exclusion could occur if this condition is absent? |
| Trigger condition for IF-WHEN | What configuration, pathway model, population, setting, or policy condition makes the requirement necessary? |
| Evidence provenance | Which evidence source supports the statement? |
| Local adaptation permitted? | Yes, no, or conditional. |
| Adaptation boundary | What must remain stable if the local form changes? |
| Reviewer or adjudicator | Who reviewed the modality assignment? |
| Disagreement or uncertainty | What was contested, and how was it resolved or deferred? |

**Worksheet B4. Evidence and Decision Agenda**

| **Field** | **Prompt** |
| --- | --- |
| Parameter | Threshold, staffing ratio, invitation rate, cost, capacity, equity effect, pathway-completion metric, referral ratio, or other parameter. |
| Why it matters | What pathway, governance, safety, equity, or resource decision depends on this parameter? |
| Why it is not fixed in the register | Why would fixing it now create false precision? |
| Evidence needed | Diagnostic accuracy study, workflow simulation, time-motion study, micro-costing, budget-impact analysis, geospatial equity analysis, cohort follow-up, implementation evaluation, or other evidence. |
| Local owner | Who is responsible for generating, reviewing, or commissioning the evidence? |
| Decision point | When will the parameter be reviewed or fixed? |
| Monitoring signal | What signal would trigger recalibration? |
| Consequence if unresolved | What risk remains if this parameter is not addressed before scale-up or adaptation? |

**Worksheet B5. Local adaptation log**

| **Field** | **Prompt** |
| --- | --- |
| Requirement being adapted | Attribute ID and requirement statement. |
| Requirement preserved? | Yes, no, or partially. |
| Local form chosen | What local delivery form is being used? |
| Reason for adaptation | Context, resources, population, venue, policy, IT, staffing, capacity, equity, acceptability, or governance constraint. |
| Risk introduced | What could fail because of the adaptation? |
| Mitigation | What control, support, or monitoring will reduce that risk? |
| Stakeholders involved | Patients/public, community partners, delivery team, receiving service, commissioner, governance lead, laboratory, primary care, or specialist service. |
| Evidence used | What local evidence, guidance, audit data, or stakeholder input supported the adaptation? |
| Review date | When will the adaptation be reviewed? |
| Decision record | Where will the decision and rationale be documented? |

**Worksheet B6. Prospective evaluation and recalibration plan**

| **Field** | **Prompt** |
| --- | --- |
| Requirement or parameter being evaluated | Attribute ID or Evidence and Decision Agenda item. |
| Evaluation question | What needs to be learned? |
| Candidate signal | What will be measured? |
| Data source | Booking system, clinical record, referral system, laboratory system, patient survey, staff log, audit, interview, observation, or community partner feedback. |
| Equity stratifier | Age, ethnicity, deprivation, language, disability, digital access, geography, comorbidity, or another locally relevant stratifier. |
| Threshold for review | What pattern would trigger adaptation, pause, or further investigation? |
| Decision authority | Who can adapt, stop, scale, or recalibrate the pathway? |
| Feedback route | How will findings be communicated to delivery teams, receiving services, community partners, and governance leads? |
| Documentation route | Where will the evaluation decision and rationale be recorded? |

**Short worked example: walk-in access**

- **Evidence excerpt or summary:** Stakeholders described walk-in access as desirable, but constrained by pre-registration, eligibility checks, staffing, space, and privacy.
- **Charted issue:** Walk-in access can improve opportunistic engagement but may create unmanaged demand, visible waiting, and privacy or throughput failures if prerequisites are not specified.
- **Requirement statement:** Specify whether the service is appointment-based, walk-in, or mixed, and align queue management, eligibility checks, staffing, and privacy controls accordingly.
- **Criticality:** IF-WHEN.
- **Criticality rationale:** Walk-in access is not a universal minimum requirement. It becomes necessary, restricted, or unsafe depending on venue, staffing, pre-registration, eligibility checks, privacy, and throughput capacity.
- **Evidence and Decision Agenda link:** If walk-in access is used, local teams should monitor invitation volume, queue length, privacy incidents or near misses, staff workload, and documentation completeness.
